# Neutrophil elastase inhibition effectively rescued angiopoietin-1 decrease and inhibits glial scar after spinal cord injury

**DOI:** 10.1186/s40478-018-0576-3

**Published:** 2018-08-07

**Authors:** Hemant Kumar, Hyemin Choi, Min-Jae Jo, Hari Prasad Joshi, Manjunatha Muttigi, Dario Bonanomi, Sung Bum Kim, Eunmi Ban, Aeri Kim, Soo-Hong Lee, Kyoung-Tae Kim, Seil Sohn, Xiang Zeng, Inbo Han

**Affiliations:** 1 Department of Neurosurgery, CHA University School of Medicine, CHA Bundang Medical Center, Seongnam-si, Gyeonggi-do 13496 Republic of Korea; 2 Department of Biomedical Science, CHA University, Seongnam-si, Gyeonggi-do Republic of Korea; 3 Molecular Neurobiology Laboratory, Division of Neuroscience, San Raffaele Scientific Institute, Milan, Italy; 4 Department of Neurosurgery, Kyung Hee University, Dongdaemun-gu, Seoul 02447 Republic of Korea; 5 College of Pharmacy, CHA University, Seongnam-si, Gyeonggi-do Republic of Korea; 6 Department of Neurosurgery, Kyungpook National University Hospital, Kyungpook National University, 130, Dongdeok-ro, Jung-gu, Daegu, 41944 Republic of Korea; 7 Department of Neurosurgery, School of Medicine,Kyungpook National University, 130, Dongdeok-ro, Jung-gu, Daegu, 41944 Republic of Korea; 8 Department of Histology and Embryology, Zhongshan School of Medicine, Sun Yat-sen University, Guangzhou, 510080 Guangdong Province China

**Keywords:** Neutrophil elastase, Spinal cord injury, Glial scar, Angiopoietins, Functional recovery, Neuropathic pain

## Abstract

After spinal cord injury (SCI), neutrophil elastase (NE) released at injury site disrupts vascular endothelium integrity and stabilization. Angiopoietins (ANGPTs) are vascular growth factors that play an important role in vascular stabilization. We hypothesized that neutrophil elastase is one of the key determinants of vascular endothelium disruption/destabilization and affects angiopoietins expression after spinal cord injury. To test this, tubule formation and angiopoietins expression were assessed in endothelial cells exposed to different concentrations of recombinant neutropil elastase. Then, the expression of angiopoietin-1, angiopoietin-2, and neutrophil elastase was determined at 3 h and at 1, 3, 5, 7, 14, 21, and 28 days in a clinically relevant model of moderate compression (35 g for 5 min at T10) spinal cord injury. A dichotomy between the levels of angiopoietin-1 and angiopoietin-2 was observed; thus, we utilized a specific neutrophil elastase inhibitor (sivelestat sodium; 30 mg/kg, i.p., b.i.d.) after spinal cord injury. The expression levels of neutropil elastase and angiopoietin-2 increased, and that of angiopoietin-1 decreased after spinal cord injury in rats. The sivelestat regimen, optimized via a pharmacokinetics study, had potent effects on vascular stabilization by upregulating angiopoietin-1 via the AKT pathway and preventing tight junction protein degradation. Moreover, sivelestat attenuated the levels of inflammatory cytokines and chemokines after spinal cord injury and hence subsequently alleviated secondary damage observed as a reduction in glial scar formation and the promotion of blood vessel formation and stabilization. As a result, hindlimb locomotor function significantly recovered in the sivelestat-treated animals as determined by the Basso, Beattie, and Bresnahan scale and footprint analyses. Furthermore, sivelestat treatment attenuated neuropathic pain as assessed by responses to von Frey filaments after spinal cord injury. Thus, our result suggests that inhibiting neutropil elastase by administration of sivelestat is a promising therapeutic strategy to inhibit glial scar and promote functional recovery by upregulating angiopoietin-1 after spinal cord injury.

**Electronic supplementary material:**

The online version of this article (10.1186/s40478-018-0576-3) contains supplementary material, which is available to authorized users.

## Introduction

Spinal cord injury (SCI) is a clinically devastating condition that can cause either temporary or permanent disability in young adults [49, 76]. SCI pathology is multifaceted. It involves several major biological cascades [48, 54, 55] and results in expeditious and enduring changes to the structure and function of microvessels [27, 56, 85], such as a loss of structural organization and microcirculation, a disruption of the blood-spinal cord barrier (BSCB), and endothelial cell (EC) and vascular remodelling [49, 85]. Vascular damage following SCI augments secondary damage, and vascular protection or the maintenance of vascular integrity mitigates this damage [30, 35, 49]. ECs participate in all facets of vascular homeostasis and play a variety of critical roles in the control of vascular functions, including in thrombosis, inflammation, and vascular wall remodeling. The death of ECs disengages the vascular network, and ischemia results in apoptosis and cell death of central nervous system (CNS) cells due to the lack of blood supply [23, 54, 55].

Recovery from SCI is preceded by angiogenesis, the extent of which correlates with neural regeneration, suggesting that angiogenesis may play a significant role in repair. Angiopoietins (ANGPTs) are vascular growth factors involved in blood vessel formation and maturation, as well as in EC survival [31, 86], and are critical regulators of vascular functions in the brain [46, 98] and spinal cord [30, 35, 74]. There are four members of the ANGPT family: ANGPT-1, − 2, and − 4 are expressed in humans, and ANGPT-3, an ortholog of ANGPT-4, is expressed in mice [31, 92]. ANGPT-1 and ANGPT-2 are released from ECs, with ANGPT-1 constitutively expressed in normal CNS vasculature [61] and ANGPT-2 weakly expressed under homeostatic conditions and increased during hypoxia, inflammation, and vascular remodeling [86]. ANGPT-1 exerts anti-inflammatory effects, reduces vessel permeability, and protects against plasma leakage in the adult vasculature [28, 87]. These effects are opposed by the actions of ANGPT-2. Therefore, it is not surprising that ANGPT-2 can antagonize the benefits to vascular integrity from endogenous or exogenous ANGPT-1 after SCI.

Neuropathological changes in spinal cord tissue result from acute inflammatory reactions that can involve elastases derived from neutrophils. Neutrophils also play a critical role in the initial events in demyelinating neuroinflammatory diseases and are intimately linked with the status of the blood-brain barrier/BSCB [7]. For example, neutrophils release a destructive proteolytic enzyme called neutrophil elastase (NE) [44]. At the vascular interface, NE induces cellular damage and dysfunction, degradation of the extracellular matrix, and pathways leading to cell death [44]. In response to proinflammatory stimuli, NE regulates the adhesion of leukocytes, clears their path for diapedesis/transmigration [62, 83, 97], and mediates the degradation of endothelial junction proteins [34, 39]. Furthermore, NE can induce apoptosis of ECs [96] and has a broad substrate specificity [69, 89]. These complex secondary pathomechanisms are responsible for extending spinal cord damage into previously uncompromised segments [66, 67, 73]. NE can induce vascular damage leading to spinal cord ischemia [84] and is also a determinant of long-term functional recovery after traumatic brain injury [81].

We hypothesized that NE might be a key determinant for the disruption/destabilization of the vascular endothelium and alter ANGPT expression after SCI. To test this, we utilized a selective NE inhibitor (sivelestat sodium; 30 mg/kg, i.p.,b.i.d.) in a rat model of moderate compression (35 g for 5 min at T10) SCI. Sivelestat attenuates NE-induced pathologies and is approved for use in patients with acute lung injury in Japan and the Republic of Korea [5, 90], and attenuates the perioperative inflammatory response in pediatric patients undergoing cardiopulmonary bypass surgery [38]. Moreover, administration of sivelestat attenuated the ischemia [41], and the chemo-attractant mRNA and protein [88] in an experimental model of SCI. However, the effect of NE inhibition on the glial scar, secondary damage, vascular stabilization, ANGPTs, ECs survival and angiogenesis after SCI remains to be determined. In the current study, we ascertain the role of NE with ANGPTs after SCI and suggest that NE inhibition endows multidimensional therapeutic strategy in tissue protection and glial scar inhibition in treating SCI.

## Material and methods

### Cell culture and treatment

In an attempt to understand the biological role of NE in ECs, we used HUVEC (ATCC) cells. HUVECs were cultured in fully supplemented endothelial growth medium as per the manufacturer instructions. Recombinant human NE protein (R&D Systems, Minneapolis, USA) was activated with 50 μg/ml Cathepsin C in assay buffer before use as per manufacturer instruction and was used at a functional concentration of 100 ng/ml, 250 ng/ml and 500 ng/ml and 1000 ng/ml, in ECs. Corning matrigel matrix was used for the tubule formation assay as per the manufacturer recommendations. Briefly, matrigel matrix was polymerized at 37 °C in a 24 well plate and HUVEC cells (passage 3) at a seeding density of 1.2 × 10 ^5^. The EGM-2 bullet kit medium were supplemented with human NE at a concentration of 100 ng/ml (group 2), 250 ng/ml (group 3), 500 ng/ml (group 4), and 1000 ng/ml (group 5). HUVEC supplemented with the only medium served as control (group 1). After 18 h, capillary-like tubules was stained with calcein AM fluorescent dye on the matrilgel. Images were randomly acquired using Cytation 3 Cell Imaging Multi-Mode Reader (Biotek Instruments,Inc., Winooski, VT, USA).

### Subjects and surgical procedures

Total 146 adult female Sprague-Dawley (SD) rats were used in the study. Rats (220–240 g) for this study were purchased from Orient Bio Inc. (Seongnam, Korea), housed in a facility at 55–65% humidity and controlled temperature of 24 ± 3 °C with light / dark cycle of 12 h, and had free access to food and water. All animal procedures were performed according to the approved protocol by the Institutional Animal Care and Use Committee (IACUC) of CHA University (IACUC160076) and Principles of laboratory animal care [63]. The animals were anesthetized with Zoletil® (50 mg/kg, Virbac Laboratories, France) / Rompun® (10 mg/kg, Bayer, Korea) solution administered intraperitoneally. Complete anesthesia was assessed using hindlimb withdrawal in response to a noxious foot pinch. After skin preparation and precise positioning of anesthetized rats, a laminectomy was performed to expose T10 spinal cord. The vertebral column was supported and stabilized by Allis clamps at T8 and T12 spinous processes as described previously [48, 75]. A metal impounder (35 g × 5 min) was then gently applied on T10 dura, resulting in moderate standing weight compression. Following compression injury, the surgical site was closed by suturing the muscle and fascia and suturing the skin; followed by external povidone-iodine application. Animals were kept on a heating pad to maintain body temperature, and then 5 mL of 0.9% sterile saline injected subcutaneously. Manual bladder expression of urine was performed twice daily until a bladder reflex was established.

### Drugs and treatments

Sivelestat sodium (Dong-A-pharma, Seoul, Korea) was dissolved in distilled water and administered twice daily intraperitoneally at the dose of 30 mg/kg 1 h after SCI; Animals were sacrificed at different time point’s day post-injury (DPI) after vehicle or sivelestat treatment. The total number of injection(s) varied based on the efficacy parameter and time points. DPI-1 (2 injections of sivelestat were given), DPI-7 (14 injections of sivelestat were given), DPI-14 (28 injections of sivelestat were given), DPI-28 (28 injections of sivelestat were given till day 14 and animals were observed till day 28 for behavioral studies and efficacy experiments).

### Pharmacokinetic study

We used SCI-injured animals for a pharmacokinetic study to correlate PK-PD. Sivelestat was treated at 1 h after SCI, The blood, brain and spinal cord samples were collected at 15 min, 30 min, 60 min, 90 min, 120 min, 150 min, 180 min after a single dose of sivelestat. For 1D, 7D, and 14 D samples, the blood, brain and spinal cord were collected after two, 14 and 28 doses of sivelestat respectively. Plasma was separated from blood using centrifugation and stored at − 80 °C until analysis. Spinal cord tissues were collected and washed with PBS and homogenized in PBS. Analysis of sivelestat in samples were performed as described in the Additional file 1.

### Behavioral assessment

#### Footprint analysis

Gait behavior and motor coordination were evaluated on 1, 7, 14, 21 and 28 days following injury, and after sivelestat treatment using the manual method as described previously with some modifications [80]. Right fore and hind paws, left fore and hind limb was painted with dyes of different colors and animals were placed over an absorbent paper surrounded by cage border. The animals were encouraged to walk in a straight line by putting a clue at the finish line. The footprint pattern was then digitalized and representative pictures were shown to assess coordination.

#### Hindlimb locomotor score

Hindlimb motor function was evaluated using the open-field Basso, Beattie, and Bresnahan (BBB) [10] locomotor test on 1, 7, 14, 21 and 28 days following injury, and after sivelestat treatment. The animals hindlimb locomotor score were evaluated by two experienced investigators who were blinded to treatment group.

#### Test for nociception

Nociception was checked using Von Frey filaments (Bioseb) as per the reported method [17]. Animals were acclimatized in rat chambers (Ugo Basile), and filaments probing were done when the animals were calm and not moving. The simplified up-down method (started with 2 g) was used to determine the mechanosensitivity/paw withdrawal threshold (PWT) with Von Frey filaments on 1, 7, 14, 21 and 28 days following injury, and after sivelestat treatment. A positive response includes flinching, licking, vocalization, or overt behavioral cue corresponding to discomfort. A total of five stimuli per test were recorded and average of three readings (lowest and highest removed) was used to determine the average PWT in Sham and after injury or treatment with sivelestat.

### qRT-PCR

Quantitative real-time PCR was carried out using an SYBR Green Master Mix and the mRNA detection was analyzed using an ABI StepOne Real-time PCR System (Applied Biosystems, Foster City, CA, USA). Primer sequences for the genes of interest and the reference gene 18S or GAPDH were as given in Additional file 2: Table S1: Typical profile times were the initial step, 95 °C for 10 min followed by a second phase at 95 °C for 15 s and 60 °C for 30 s for 40 cycles with a melting curve analysis. The target mRNA level was normalized with the level of the 18S or GAPDH and compared with the control. Data were analyzed using the ΔΔCT method.

### Western blot analysis

Spinal cord tissues were collected and washed with PBS, placed at 4^∘^ C, and homogenized using T 25 digital homogenizer (IKA, Seoul, Korea) in lysis buffer (1× RIPA lysis buffer) and then finally passed through a 31^1/2^ gauge syringe needle and centrifuged at 14,000 rpm at 4^∘^C for 15 min. Protein concentration was determined in supernatants using Bio-Rad DC Protein Assay (Hercules, CA, USA). Equal amounts (40 μg) of protein were separated electrophoretically by 10% SDS-PAGE electrophoresis, and the resolved proteins were transferred to PVDF membranes (Millipore, Bedford, MA, USA). The membranes were incubated for 1 h with 5% non-fat skim milk prepared in TBS buffer to block nonspecific binding. The membranes were then incubated overnight with primary antibodies to ANGPT-1 (1:10000, Abcam, Cambridge, UK), ANGPT-2 (1:1000, Abcam), NE (1;1000), ZO-1 (1:500, Invitrogen, California, USA), AKT (1:1000, Cell Signaling Technology, Danvers, MA, USA), pAKT (1:1000, Cell Signaling Technology,) LC3B (1:1000, Cell Signaling Technology,), and Actin (1:10000, ABM). After 1-h incubation with corresponding secondary antibodies, The blots were visualized with a PowerOpti-ECL (Animal Genetics Inc., Gainesville, FL, USA) detection system, according to the recommended procedure. Immunoreactivity was detected using the BIORAD ChemiDoc™ XRS.

### Immunohistochemistry and immunofluorescence

At 1, 7, 14 and 28 days after compression of the spinal cord at T10, animals were anesthetized with mixture of Zoletil® (50 mg/kg, Virbac Laboratories, France) / Rompun® (10 mg/kg, Bayer, Korea) solution administered intraperitoneally and perfused with 0.9% saline followed by 4% paraformaldehyde for tissue fixation. The spinal cord at the compression site was removed, and immersed in 4% paraformaldehyde for 1 day, and then embedded in paraffin, sectioned at 5 or 10 μm, dewaxed, and stained with antibodies against ANGPT-1 (1:500, Abcam, Cambridge, UK), ANGPT-2 (1:200, Abcam), N.E. (1;50, Abcam), GFAP (1:1000, Abcam), GFAP (1:200, Sigma), Iba-1 (1:200, Abcam) ZO-1 (1:50, Invitrogen, California, USA), Occludin (1:50, Invitrogen), RECA (1:100, Abcam), TGF-β1 (1:100, Abcam), NG-2 (1:500, Millipore), α-SMA (1:500, Abcam)PECAM(1:100, Abcam), Caspase-3 (1:50, Abcam), CD68 (1:200, Abcam), Arginase (1:100, Abcam), Laminin (1:50, Abcam), BDNF (1:200, Alomone Lab), NT-3 (1:200, Alomone Lab), NT-4 (1:200, Alomone Lab), vWF (1:50, Abcam), NF-200 (1:1000, Abcam), Tuj-1 (1:200, Abcam), VEGF (1:100, Abcam) TNF-α (1:50, Abcam), Fibronectin (1:50, Abcam), RECA-1 (1:100, Abcam), IL-6 (1:50, Abcam). Secondary antibodies (1:200 or 1:500) were goat anti-rabbit Alexa Fluor^®^ 488 (Abcam), goat anti-rabbit Alexa Fluor^®^ 647 (Abcam), goat anti-rabbit Alexa Fluor^®^ 568 (Abcam), goat anti-rabbit Alexa Fluor^®^ 555 (Abcam), goat anti-mouse Alexa Fluor^®^ 488 (Abcam), goat anti-mouse Alexa Fluor^®^ 568 (Abcam), chicken-anti-goat 647 (Invitrogen).Following washing after secondary antibody incubation DAPI (1:500) was incubated for 10 min. Sections were mounted and examined using a fluorescence microscope (Zeiss, Oberkochen, Germany or Leica, Germany).

### Statistical analysis

All data were analyzed using Graph Pad Prism ver. 5.01 (Graph Pad, Inc., La Jolla, CA, USA). All data are expressed as mean ± SEM. One-way ANOVA was performed and Dunnett’s posthoc test was used to analyze the in-vitro data. The in-vivo PCR data was analyzed using One-way ANOVA followed by Tukey’s test. The BBB scores were analyzed statistically with Kruskal-Wallis test at each time point. The nociception data were analyzed using Two-way ANOVA followed by Bonferroni test. *P-*values < 0.05 were considered statistically significant.

## Results

### NE suppresses capillary-like tubule formation and ANGPT expression in ECs, whereas proinflammatory factors differentially modulate ANGPT expression

**Fig. 1:**
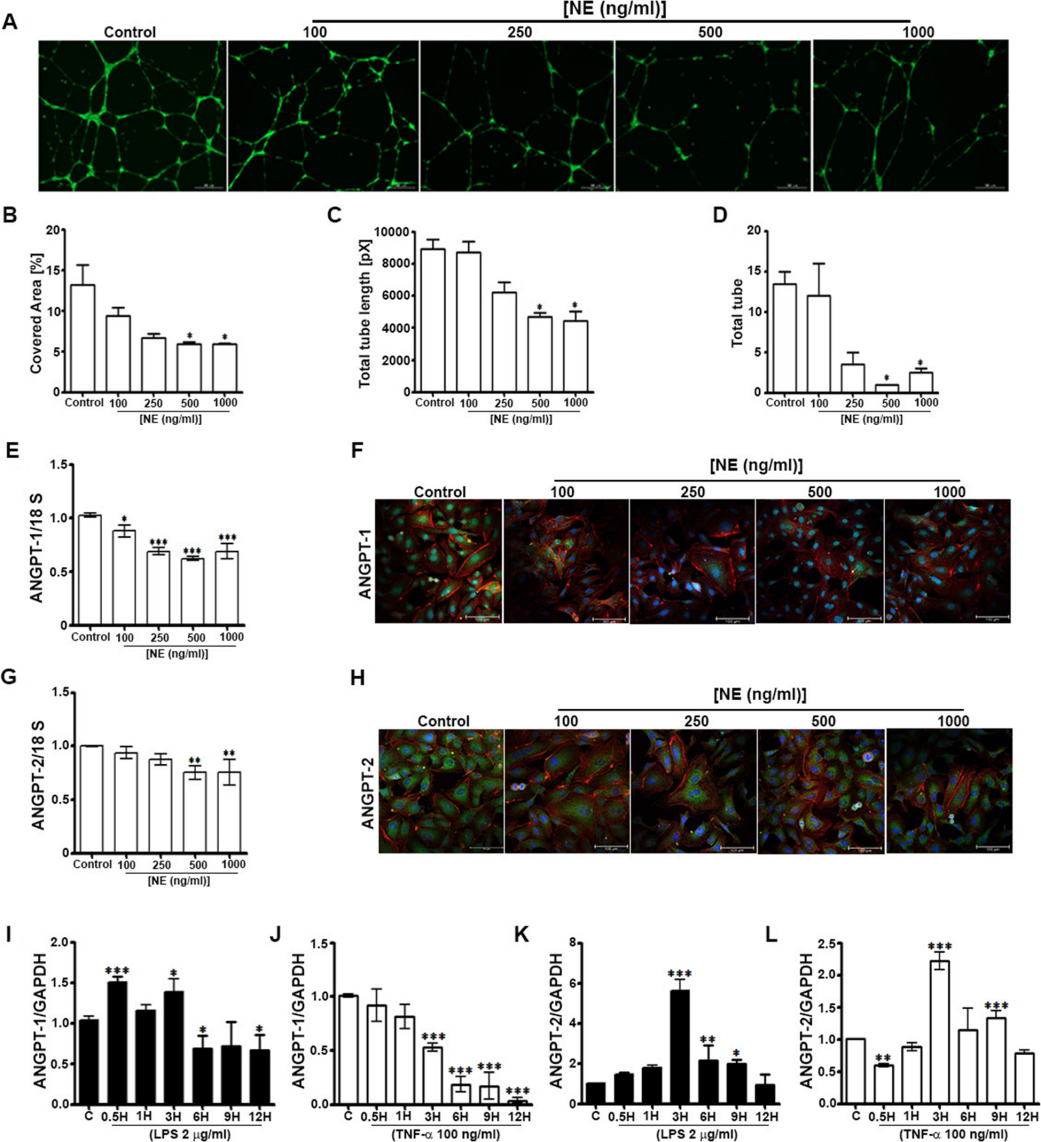
Neutrophil elastase (NE) impedes tubule formation and decreases angiopoietin (ANGPT) expression, whereas inflammatory factors differentially modulate ANGPT expression, in human umbilical vein endothelial cells (HUVECs). A tubule formation assay was performed as described in the Methods section. Recombinant human NE was added at concentrations of 100, 250, 500, and 1000 ng/ml (HUVECs exposed only to medium served as the control) to determine tubule formation (**a**), the percentage of covered area (**b**), total tube length (**c**), and total numbers of tubes (**d**). **e** and **g** Total RNA was prepared from HUVECs exposed to various concentrations of NE for 24 h to determine the expression of *ANGPT1* and *ANGPT2*. **f** and **h** ANGPT-1 and ANGPT-2 immunocytochemistry was performed on fixed HUVECs as described in the Methods sections. **i–l** *ANGPT1* and *ANGPT2* mRNA expression was determined by real-time quantitative reverse transcription-polymerase chain reaction in HUVECs collected 0.5, 1, 3, 6, 9, and 12 h after treatment with lipopolysaccharide ([LPS] 2 μg/ml) and tumor necrosis factor alpha ([TNF-α] 100 ng/ml). 18S was used as the internal control. Data represent means ± SEMs (*n* = 2–3/group performed in triplicate). *p < 0.05, **p < 0.01, ***p < 0.001 vs. control

We used various strategies to elucidate the effects of NE and inflammation on ANGPT expression in ECs. First, we determined the effects of recombinant human NE protein (100, 250, 500, and 1000 ng/ml) on human umbilical vein ECs (HUVECs); HUVECs treated with medium only served as the control. The results of a tubule formation assay showed a dose-dependent decrease in the tubule-covered area, total tube length, and total numbers of tubes, with significant effects observed at NE concentrations of 500 ng/ml and 1000 ng/ml (Fig. 1a–d).The expression of ANGPT-1 and ANGPT-2in HUVECs treated with NE for 24 h was determined by RT-PCR and immunocytochemistry. The expression of ANGPT-1 was dose-dependently decreased following the addition of NE (Fig. 1e and f), and ANGPT-2 expression decreased at higher doses (500 ng/ml and 1000 ng/ml) (Fig. 1g and h). Additionally, the effect of inflammation on ANGPT expression was assessed by treating HUVECs with lipopolysaccharide ([LPS] 2 μg/ml) and tumor necrosis factor alpha ([TNF-α] 100 ng/ml). ANGPT-1 expression increased at 0.5 and 3 h and decreased at 6, 9, and 12 h after the addition of LPS to the medium (Fig. 1i), whereas expression was significantly decreased by TNF-α at 3–12 h (Fig. 1j). By contrast, ANGPT-2 expression increased at 3, 6, and 9 h after the addition of LPS (Fig. 1k) and increased at 3 and 9 h after TNF-α was added (Fig. 1l). These results suggest that NE and inflammation differentially modulate the expression of ANGPTs in ECs.

### SCI disrupts vascular endothelial integrity and alters NE and ANGPT expression

**Fig. 2:**
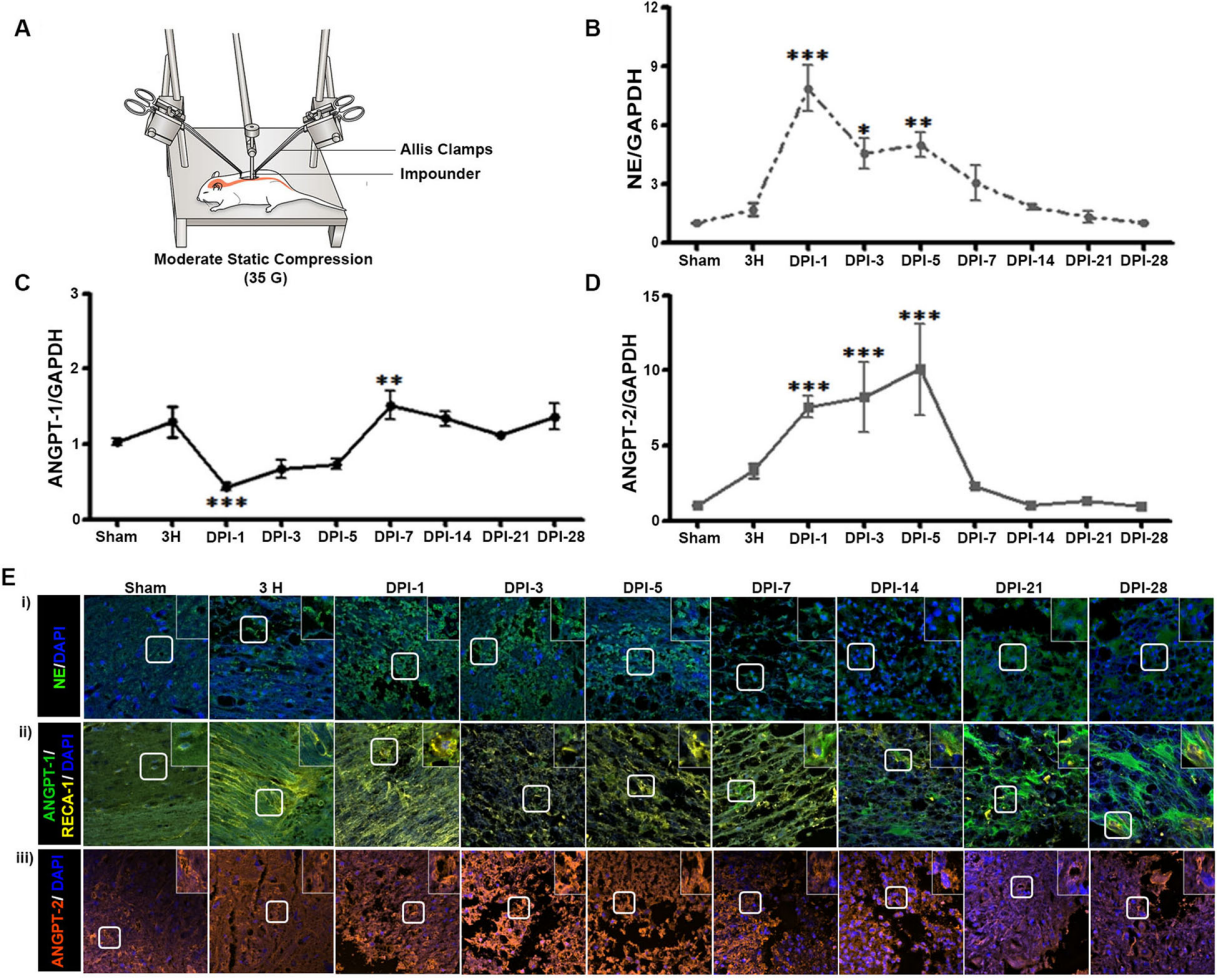
Neutrophil elastase (NE) and angiopoietin-2 (ANGPT-2) expression increases and angiopoietin-1 (ANGPT-1) and rat endothelial cell antigen (RECA-1) expression decreases after spinal cord injury (SCI) at the epicenter of the damage. **a** Schematic showing SCI method. Total RNA was prepared from spinal cord tissues at the epicenter of the damage collected 3 h and 1, 3, 5, 7, 14, 21, and 28 days after SCI to determine the expression of NE (**b**), ANGPT-1 (**c**), and ANGPT-2 (**d**). **e** Representative images of immunohistochemistry performed on longitudinal sections for NE (i), ANGPT-1 and RECA-1(ii), and ANGPT-2 (iii) at different time points after SCI [3 fields/slide, *n* = 2–3/group (sham = 2, and injury = 3)]. GAPDH was used as internal controls for real-time quantitative reverse transcription–polymerase chain reaction. Data represent means ± S.E.M. [*n* = 2–3/group (sham = 2, and injury = 3) performed in triplicates]. *p < 0.05, **p < 0.01, ***p < 0.001 compared with Sham group

We characterized the time course of NE, ANGPT-1, and ANGPT-2 mRNA and protein expression at the epicenter of the damaged spinal cords in rats at 3 h and 1, 3, 5, 7, 14, 21, and 28 days after moderate compression injury (35 g for 5 min) (Fig. 2a).There was a significant increase in NE expression from 3 h to 5 days after SCI, with maximum expression observed at 1 day after SCI (Fig. 2b and ei). A dichotomy was observed between ANGPT-1 and ANGPT-2 expression patterns. The expression of ANGPT-1 was initially drastically reduced (the maximum decrease was observed 1 day after SCI when NE expression was maximal) and then increased at 7 days after SCI and remained elevated (Fig. 2c and eii), whereas the expression of ANGPT-2 continuously increased through 5 days after SCI and unexpectedly decreased at 7 days, when the ANGPT-1 expression was increased, and consequently returned to normal (Fig. 2d and eiii). As ANGPTs are expressed primarily by ECs, we determined the integrity of the vascular endothelium using immunohistochemistry (IHC) with rat EC antigen ([RECA-1] Fig. 2eii), which revealed progressive damage after SCI. RECA-1-stained vessels were readily identified within the injured spinal cord.

### Sivelestat increases ANGPT-1 and decreases ANGPT-2 and NE expression after SCI

**Fig. 3:**
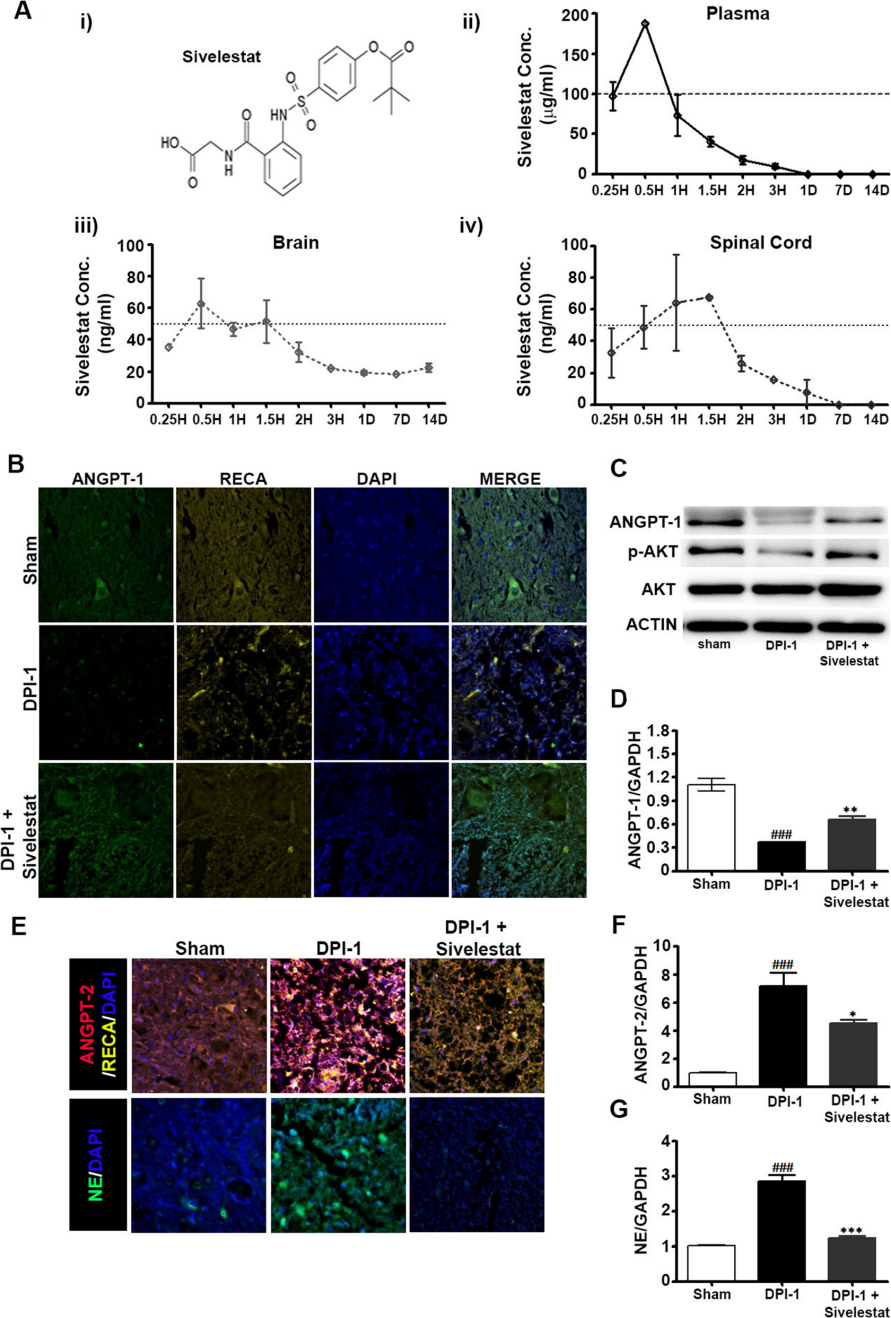
Neutrophil elastase (NE) inhibition via sivelestat prevented the spinal cord injury (SCI)-induced modulation of angiopoietins (ANGPTs) and inhibited the expression of NE. **a** Molecular structure of sivelestat (i) and concentrations determined in plasma (ii), brain (iii), and spinal cord (iv) at several time points as described in the Methods section (*n* = 2/timepoint). Samples from sham, injured untreated, and injured sivelestat-treated animals were prepared DPI-1 as described in the Methods section. Representative images of immunohistochemistry for ANGPT-1 and rat endothelial cell antigen (RECA-1) (**b**) and ANGPT-2 and NE (**e**) at 1 day after SCI [3 fields/slide, *n* = 2–3/group (sham = 2, injury = 3 and sivelestat = 3)]. **c** Western blots of ANGPT-1, p-AKT, and AKT expression at 1 day after injury. Actin was used as internal controls for western blot [*n* = 2–3/group (sham = 2, injury = 3 and sivelestat = 3)]. Total RNA and spinal extracts from sham or injured untreated (Injury) or after sivelestat treatment were prepared 1 day after the injury. RT-PCR results of Ang-1 (**d**), Ang-2 (**f**) and N.E (**g**) expression 1 day after injury [*n* = 2–3/group (sham = 2, injury = 3 and sivelestat = 3) performed in triplicates]. GAPDH was used as internal controls for real-time quantitative reverse transcription–polymerase chain reaction. Data represent means ± S.E.M. ^###^*p* < 0.001 vs. sham group. *p < 0.05, **p < 0.01, ***p < 0.001 vs. Injury group

The data above indicated that peak expression of NE occurred 1 day after SCI, accompanied by increased ANGPT-2 and decreased ANGPT-1 expression. Therefore, we determined the effect of inhibiting NE on ANGPT expression at DPI-1. One group of animals was treated with sivelestat (30 mg/kg, i.p., b.i.d.), a specific inhibitor of NE, and the concentrations in plasma, brain, and spinal cord were monitored over 14 days (Fig. 3a). Samples from sham, injured untreated, and injured sivelestat-treated (two doses) animals were prepared on DPI-1. Interestingly, sivelestat treatment prevented the SCI-induced decrease in ANGPT-1 expression (Fig. 3b–d) and attenuated the SCI-induced increase in ANGPT-2 at DPI-1 (Fig. 3e and f). Treatment with sivelestat also significantly reduced NE expression at DPI-1 (Fig. 3e and g). Additionally, the phosphorylation of AKT (p-AKT) was reduced in the spinal cord following SCI, which was prevented by treatment with sivelestat (Fig. 3c).

### NE inhibition attenuates the expression of inflammatory cytokines and chemokines after SCI

**Fig. 4:**
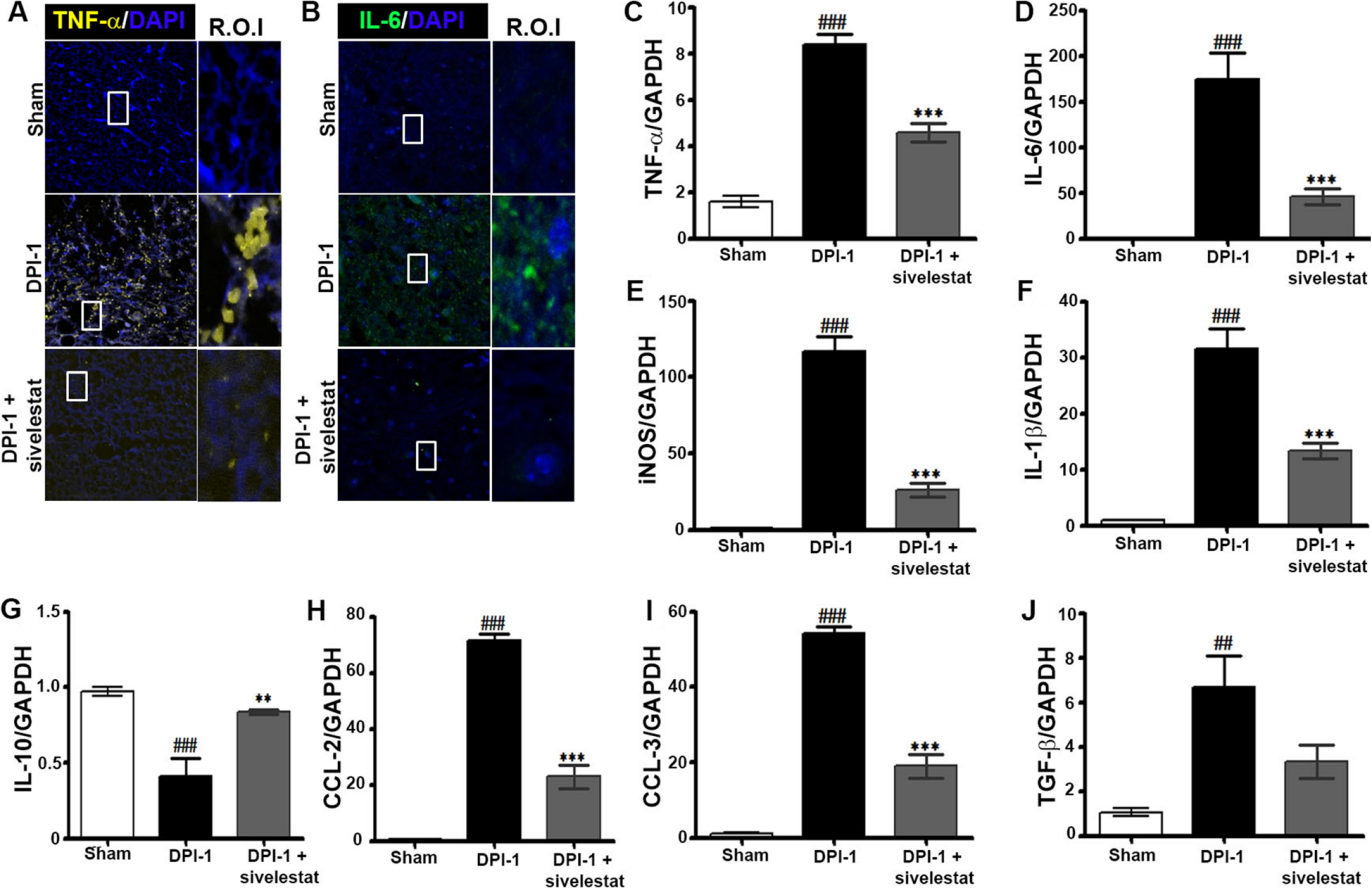
Neutrophil elastase (NE) inhibition reduces the expression and production of inflammatory mediators (cytokines and chemokines) after spinal cord injury (SCI). Samples from sham or injured untreated (Injury) or after sivelestat treatment were prepared 1 day after injury as described in the Methods section. Representative section of tumor necrosis factor-alpha [TNF-α (**a**)], Interleukin [IL-6 (**b**)] immunofluorescence 1 day after SCI [3 fields/slide, *n* = 2–3/group (sham = 2, injury = 3 and sivelestat = 3)]. Total RNA from sham, vehicle (injury)- or sivelestat-treated samples were prepared DPI-1 after injury as described in the Methods section. RT-PCR results showing relative expression levels of TNF-α (**c**), IL-6 (**d**), inducible nitric oxide synthase [iNOS (**e**)], IL-1β (**f**), IL-10 (**g**), chemokine (C-C motif) ligand 2 [CCL-2 (**h**)], chemokine (C-C motif) ligand 3 [CCL-3 (**i**)] and TGF-β (**j**) after injury/treatment [*n* = 2–3/group (sham = 2, injury = 3 and sivelestat = 3) performed in triplicates]. GAPDH was used as internal controls for real-time quantitative reverse transcription–polymerase chain reaction. Data represent means ± S.E.M. ^##^*p* < 0.01, ^###^*p* < 0.001 vs. sham group. *p < 0.05, **p < 0.01, ***p < 0.001 vs. Injury group

We and others have shown that the peak expression of inflammatory markers (cytokines and chemokines) occurs in the acute phase of SCI [48]. Therefore, we determined the effect of NE inhibition on inflammatory parameters using spinal cord samples from sham, injured untreated (Injury), and injured sivelestat-treated (two doses) animals prepared on DPI-1. Sivelestat treatment significantly attenuated the SCI-induced expression of TNF-α (Fig. 4a and c) and interleukin (IL)-6 (Fig. 4b and d).Similarly, the induction of the inflammatory mediators inducible nitric oxide synthase (iNOS) and IL-1β was also significantly decreased with sivelestat (Fig. 4e and f, respectively). Interestingly, sivelestat reversed the suppression of the anti-inflammatory cytokine IL-10 (Fig. 4g) and significantly reduced the SCI induction of C-C motif chemokine ligand (CCL)-2 and CCL-3 (Fig. 4h and i, respectively) and TGF-β (Fig. 4j).

### NE inhibition attenuates TJ disruption and blood-spinal cord permeability after SCI

**Fig. 5:**
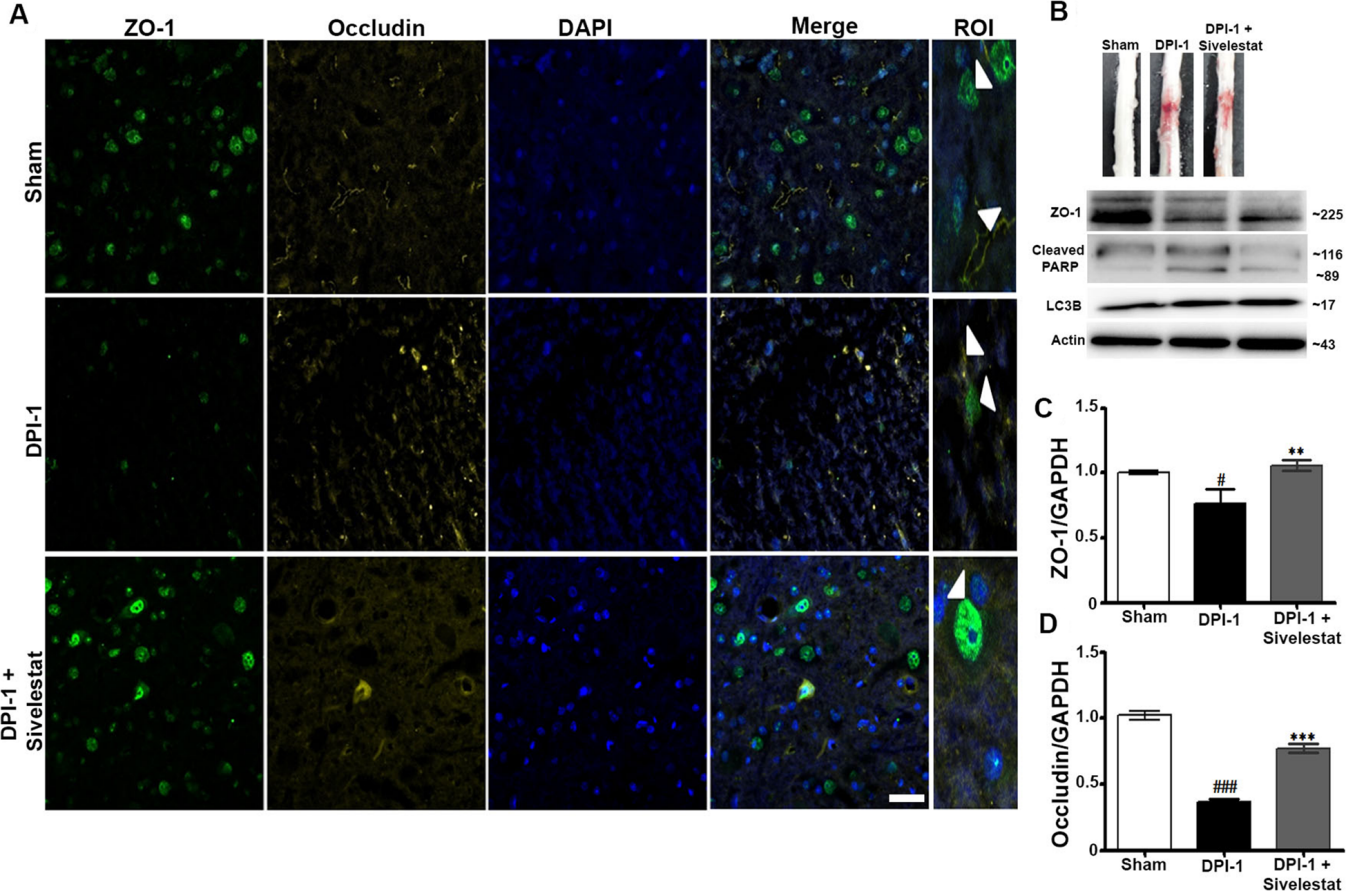
Neutrophil elastase inhibition prevented the spinal cord injury (SCI)-induced disruption of tight junction protein (ZO-1 and Occludin) in a rat model. Samples from sham, injured untreated, and injured sivelestat-treated animals were prepared DPI-1 as described in the Methods section. **a** Representative section of zonula occludens (ZO)-1 and occludin immunofluorescence, at DPI-1[3 fields/slide, *n* = 2–3/group (sham = 2, injury = 3 and sivelestat = 3)]. **b** Western blots of ZO-1, cleaved PARP, and LC3B expression at 1 day after injury. Actin was used as internal controls for western blot. Total RNA and spinal extracts from sham or injured untreated (Injury) or after sivelestat treatment were prepared DPI-1 as described in the Methods section. RT-PCR results of occludin and zonula occludens (ZO)-1 expression 1 day after injury [*n* = 2–3/group (sham = 2, injury = 3 and sivelestat = 3) performed in triplicates] (**c** and **d**). GAPDH was used as internal controls for real-time quantitative reverse transcription–polymerase chain reaction. Data represent means ± S.E.M. ^#^*p* < 0.05, ^###^*p* < 0.001 vs. sham group. **p < 0.01, ***p < 0.001 vs. Injury group

The disruption of the BSCB and subsequent blood infiltration after SCI initiates a secondary injury cascade via the production of inflammatory mediators, such as IL-6, TNF-α, and iNOS. To establish the role of NE in BSCB disruption, we examined samples from sham, injured untreated, and injured sivelestat-treated (two doses) animals prepared DPI-1. NE inhibition via sivelestat administration significantly inhibited the loss of the tight junction (TJ) proteins occludin and zonula occludens-1 (ZO-1) after injury (Fig. 5a–d). SCI induced haemorrhage which was reduced by sivelestat treatment, and expression of cleaved PARP and LC3B was also decreased following treatment (Fig. 5b).

### NE inhibition attenuates secondary damage and prevents glial scar formation after SCI

**Fig. 6:**
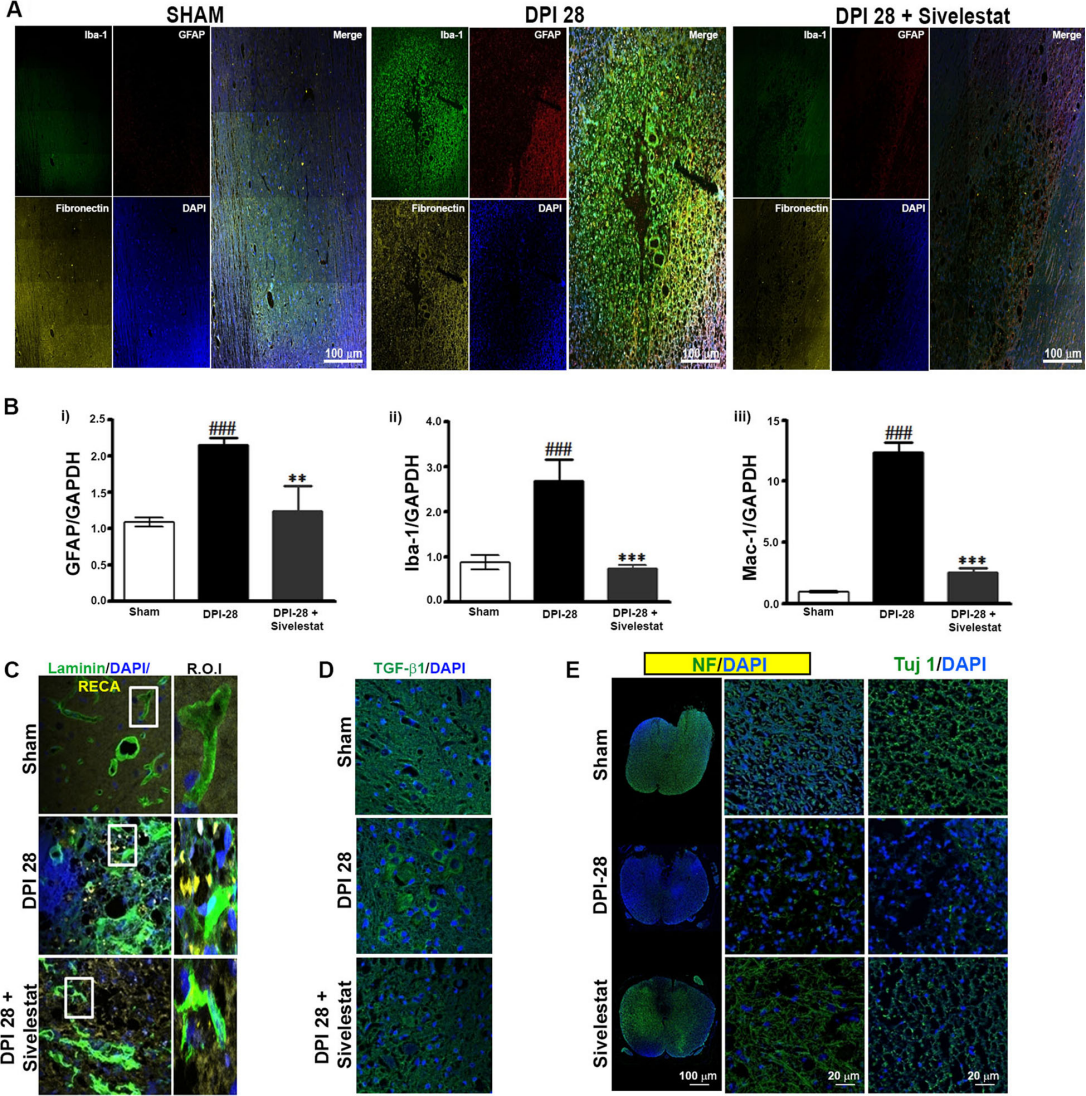
Neutrophil elastase inhibition attenuates inhibitory glial/fibrotic scarring and secondary damage after spinal cord injury (SCI). Samples from sham or injured untreated (Injury) or after sivelestat treatment were prepared at DPI-28 as described in the Methods section. Sivelestat (30 mg/kg, i.p.) was given for 14 days (28 doses) and animals were sacrificed at day 28. Zeiss confocal microscope was used to evaluate the Immunofluorescence after IHC. The merge function of Zeiss microscope was used and 16 areas were evaluated. **a** Representative merges images (longitudinal) for Iba-1 (green), GFAP (red), fibronectin (yellow) at DPI-28. RT-PCR results of GFAP (**b**-i), Iba-1 (**b**-ii), and Mac-1 (**b**-iii), expression at DPI-28 (*n* = 3/group). Representative images of laminin (green), rat endothelial cell antigen (RECA-1; yellow) (**c**), TGF-β1 (green) (**d**) and neurofilaments (N.F) and Tuj-1 (**e**), at DPI-28 (3 fields/slide, *n* = 3/group). GAPDH was used as internal controls for real-time quantitative reverse transcription–polymerase chain reaction. Data represent means ± S.E.M. ^###^*p* < 0.001 vs. sham group. **p < 0.01, ***p < 0.001 vs. Injury group

Fibrotic scar tissue is rich in microglia, astroglia, and laminin, and fibronectin forms at the lesion site after SCI in rodents and humans [77]. Several axon-inhibitory molecules present at this scar tissue facade hinder axon regeneration. To evaluate the role of NE in secondary damage and glial scar formation, we examined samples from sham, injured untreated, and injured sivelestat-treated (for 14 days) animals prepared 28 days after SCI (DPI-28). We observed decreases in secondary damage and glial scar formation in animals treated with sivelestat, which were associated with substantial inhibitory effects observed via IHC for microglia (Iba-1), astroglia (GFAP), and fibronectin (Fig. 6a) at the injury site. The decreases in astroglial and microglial activation, as well as macrophage activation, were confirmed at the transcriptional level via quantitative reverse transcription-PCR analysis (Fig. 6bi–iii). IHC for laminin showed a J- or T-shaped morphology under normal conditions, whereas fibrotic scars rich in laminin formed at the epicenter after SCI (Fig. 6c). Treatment with sivelestat attenuated the laminin expression at DPI-28 as well as SCI-induced transforming growth factor beta (TGF-β) expression (Fig. 6d). IHC for the neuronal marker TuJ1 and the axonal marker neurofilament (NF) revealed significant regeneration in the lesions of sivelestat-treated rats (Fig. 6e).

### NE inhibition stabilizes vascular endothelium formation after SCI

**Fig. 7:**
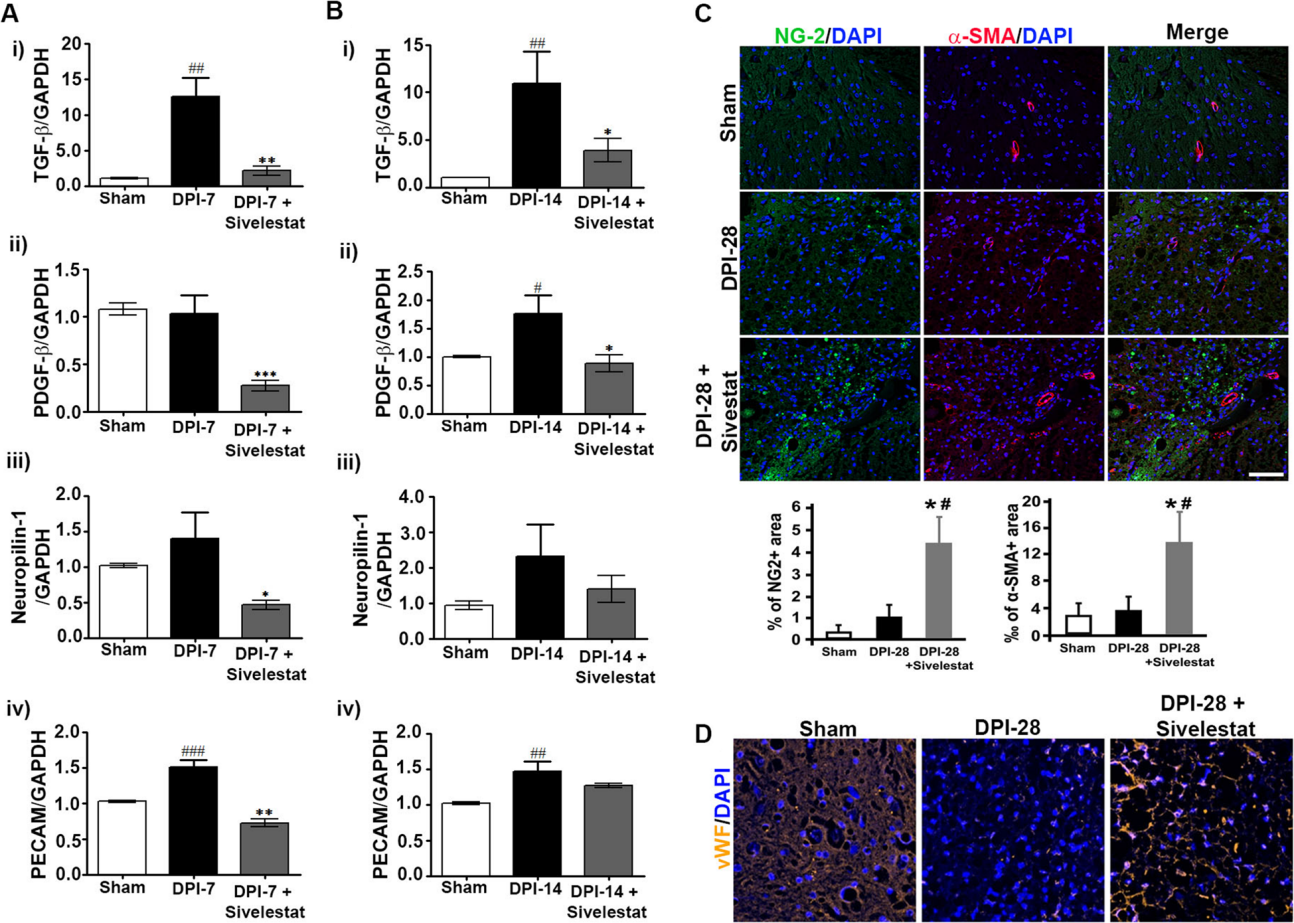
Neutrophil elastase inhibition stabilizes vascular endothelium after spinal cord injury (SCI). Samples from sham or injured untreated (Injury) or after sivelestat treatment were prepared 7 or 14 or 28 day after injury as described in the Methods section. Total RNA from sham, vehicle (injury) - or sivelestat-treated samples were prepared 7 and 14 day after injury as described in the Methods section. RT-PCR results showing relative expression levels of Transforming growth factor-β1 [TGF-β1; **a** and **b** (i)], Platelet-derived growth factor [PDGF; **a** and **b** (ii)], Neuropilin-1 [**a** and **B** (iii)], and platelet endothelial cell adhesion molecule [PECAM; **a** and **b** (iv)] [*n* = 2–3/group (sham = 2, injury = 3 and sivelestat = 3) performed in triplicates]. Representative section of neural/glial antigen [NG-2 (**c**); green], alpha-smooth muscle actin [α- SMA(**c**) red]; Von Willebrand factor [vWF (**d**) orange] after SCI. Bar charts show the percentage of NG2 or α-SMA positive area per randomly selected field (3 fields/slide, *n* = 3/group) (**c**). GAPDH was used as internal controls for real-time quantitative reverse transcription–polymerase chain reaction. Data represent means ± S.E.M. ^#^*p* < 0.05, ^##^*p* < 0.01, ^###^*p* < 0.001 vs. sham group. **p < 0.01, ***p < 0.001 vs. Injury group

SCI substantially disrupts the vasculature, especially at the epicenter of the injury, where blood vessels undergo remodeling and structural changes before they become functional. Therefore, we delineated the changes associated with vascular-related proteins and factors after SCI in untreated and sivelestat-treated animals. SCI-induced increases in TGF-β, platelet-derived growth factor beta (PDGF-β), neuropilin1, and platelet-endothelial cell adhesion molecule (PECAM), indicative of vascular changes/damage and vascular remodeling. In contrast to those of untreated (vehicle-injected) animals, the spinal cords of sivelestat-treated animals at 7 and 14 days after SCI exhibited an attenuation of these increases in vascular-related factors and a stabilization of the vasculature (Fig. 7a and b). IHC of spinal cord microvessels also revealed a change in the expression of neural/glial antigen2 (NG-2), alpha-smooth muscle actin (α-SMA) (Fig. 7c), and von Willebrand factor (vWF) (Fig. 7d). Treatment with sivelestat increases the expression of NG-2, α-SMA and vWF.

### NE inhibition improves functional recovery, attenuates nociception, and provides neuroprotection after SCI

**Fig. 8:**
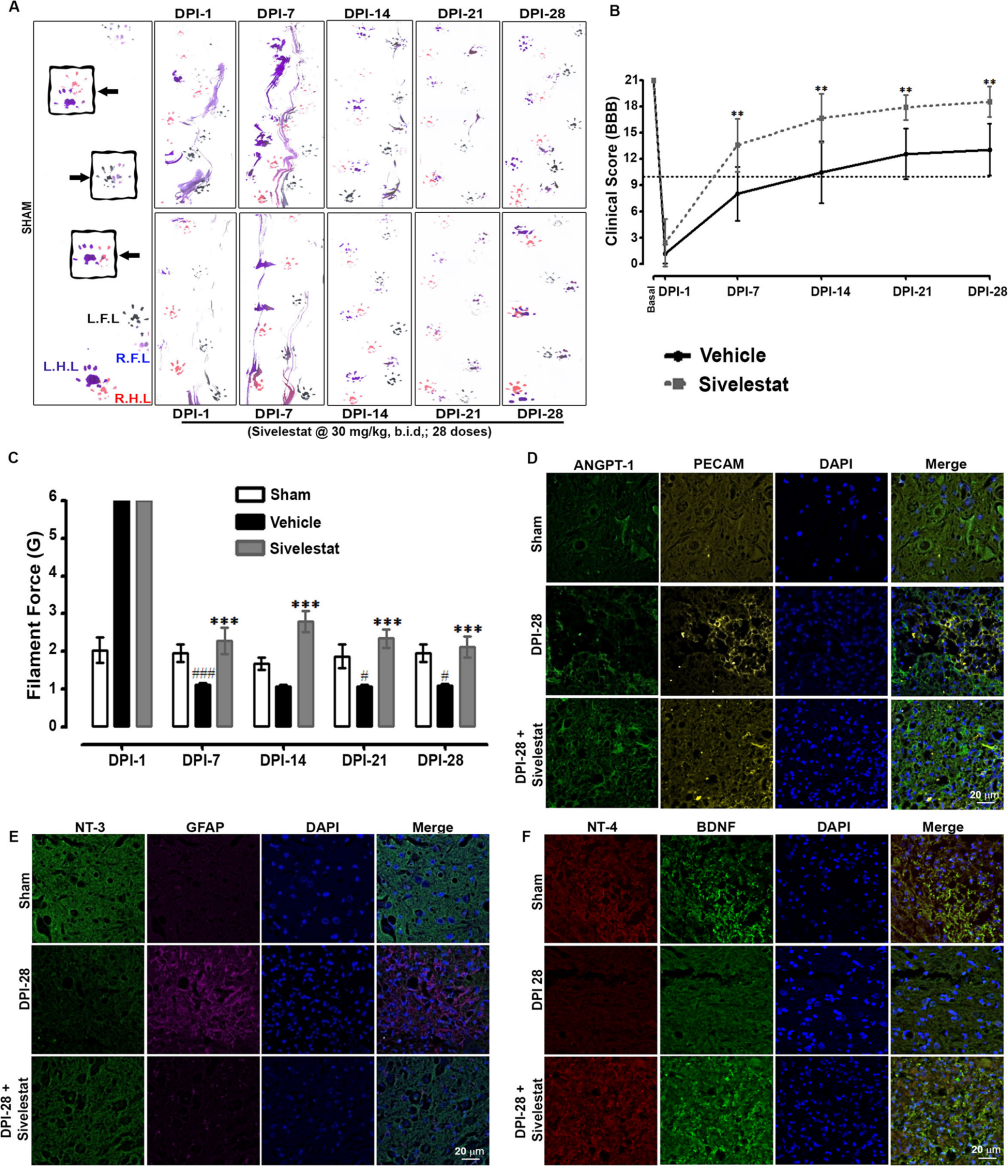
Sivelestat treatment improved motor functions and attenuated neuropathic pain after spinal cord injury (SCI). **a** Representative footprints of animal walking 1, 7, 14, 21 and 28 days after SCI. Black: Left forepaw footprints; Blue: Right forepaw footprints; Purple: left hind paw footprints; Red: Right hind paw footprints. **b** Functional recovery was then assessed in open-field testing by using the 21-point Basso, Beattie, and Bresnahan (BBB) locomotor test at 1, 7, 14, 21 and 28 days after SCI. **c** Nociception was evaluated using Von Frey-filaments; SCI-induced hypersensitivity (decrease in withdrawal threshold) was assessed at 1, 7, 14, 21 and 28 days after SCI (sham = 6, injury = 16 and sivelestat = 12 for behavioral experiments). Representative sections of ANGPT-1 and PECAM (**d**), neurotrophin-3 (NT-3) and Glial fibrillary acidic protein (GFAP) (**e**), and neurotrophin-4 (NT-4) and Brain-derived neurotrophic factor (BDNF) (**f**), at DPI-28. Data represent means ± S.E.M. ^#^*p* < 0.05, ^###^*p* < 0.001 vs. sham group. ***p < 0.001 vs. Injury group

We performed behavioral tests at 1, 7, 14, 21, and 28 days following SCI on untreated animals and animals treated for 14 days with sivelestat. Specifically, walking patterns (gait) were evaluated via manual analyses of footprints. After SCI, all animals showed distinct reductions in motor coordination in forepaw-hindpaw stepping. Those treated with sivelestat showed a marked recovery of gait and improved motor coordination in comparison with untreated animals (Fig. 8a). The performance of sham rats remained unchanged throughout the testing period. Functional recovery was also assessed in open-field testing using the 21-point Basso, Beattie, and Bresnahan (BBB) locomotor test [10]. Animals developed paraplegia after SCI, corresponding to a low BBB score, but showed evidence of modest improvements of motor function as early as 3–4 days after injury (Fig. 8b). Recovery then continued reasonably faster for another 1–2 weeks and then subsequently proceeded at a slower rate. The recovery in motor function was significantly faster in animals treated with sivelestat than in untreated animals. Interestingly, BBB scores were significantly higher in the sivelestat group even after the treatment had stopped (i.e., after 14 days). As nociception is one of the most common devastating conditions after SCI, we evaluated the effect of sivelestat on SCI-induced pain using vonFrey filaments. After SCI, animals exhibited tactile hypersensitivity (decrease in withdrawal threshold) on days 7, 14, 21, and 28, but not at day 1 (Fig. 8c). Treatment with sivelestat significantly attenuated the SCI-induced hypersensitivity.

Finally, the expression of neurotrophic factors, which are mediators of neuronal and axonal plasticity and regeneration after SCI, was examined by IHC. The expression of brain-derived neurotrophic factor (BDNF), ANGPT-1, neurotrophin3 (NT-3), and neurotrophin4 (NT-4) declined, whereas the expression of GFAP increased, after SCI (Fig. 8d, g and f). Consistent with the other findings in this study, sivelestat treatment prevented the decrease in BDNF, ANGPT-1, NT-3, and NT-4 and attenuated the increase in GFAP expression.

## Discussion

In the present study, we demonstrate that NE is a key determinant of vascular endothelium disruption/destabilization and affects ANGPT expression in vitro (ECs) and in a rat model of SCI. First, the effects of various concentrations of recombinant NE were assessed on the most-characterized type of ECs, HUVECs. ECs form capillary-like structures in response to angiogenic factors present in the growth medium; however, the addition of NE recombinant protein dose-dependently prevented the formation of capillary-like tubules, reducing the total length and numbers of tubes. These findings suggest that NE influences a required step in the process of angiogenesis. NE also suppressed the expression of ANGPT-1 and ANGPT-2 in ECs. The reduced expression of these factors may explain the decreased tubule formation in ECs. We also found that factors known to induce inflammation (i.e., LPS and TNF-α), which impact SCI and ANGPT in ECs [26, 48, 49], decreased the expression of ANGPT-1 and increased that of ANGPT-2, suggesting that NE and inflammation differentially modulate the expression of ANGPTs in ECs.

The expression patterns of NE and ANGPTs and the roles of NE inhibition in neuroinflammation, BSCB disruption, vascular damage, functional recovery, and neuroprotection after SCI were also examined. The compression injury used in this study results in damage to both the dorsal and ventral spinal cord, transient ischemia, and impaired blood flow, which act synergistically to promote secondary pathology as typically observed in SCI in humans. Neutrophil infiltration is linked to progressive damage to the BSCB after SCI, and the prevention of this promotes functional recovery [52, 64]. In the present study, the expression of NE increased significantly from 3 h to 5 days, with maximal expression observed at DPI-1. We also observed damage to the vascular endothelium at this time point via IHC for RECA-1. Since the release of NE from neutrophils can damage ECs, we surmised that NE can also affect the proteins released from ECs. Interestingly, ANGPT-1 expression was downregulated when the expression of NE was maximal and corresponded to the initial progressive damage to the endothelium. From day 7 on, ANGPT-1 expression increased, which may be due to expression in a subset of glial and neuronal cells [13]. ANGPT-2 sensitizes ECs to inflammation, enhancing vascular responsiveness to proinflammatory cytokines [26]. ANGPT-1 and ANGPT-2 have similar affinities for the same receptor but produce opposing effects on blood vessels. SCI induces a lasting decrease in ANGPT-1 levels [14, 35, 58, 74] but a persistent upregulation of ANGPT-2 [22]. Indeed, we observed an increase in ANGPT-2 expression that may counterbalance the ANGPT-1 decrease after SCI. These findings suggest that SCI-induced NE expression can cause endothelium damage and modulate the activity of ANGPT-1 activity after SCI.

To examine this, we used sivelestat, a specific inhibitor of NE. The pharmacokinetic results in the present study show that the concentration of sivelestat in blood peaks at around 30 min, with no accumulation, observed even after 28 doses, indicating that the drug is cleared from the body. Sivelestat treatment significantly attenuated the SCI-induced NE and ANGPT-2 expression, reduction in ANGPT-1, and endothelial damage. These results suggest that specific inhibition of NE prevents the modulation of ANGPT, restores SCI-induced endothelial damage, and prevents vascular disruptions.

SCI is accompanied by a series of intense immune responses, including inflammation, the synthesis of chemokines and cytokines, and the infiltration of peripheral leukocytes to the site of damage. Sivelestat was shown to prevent neutrophil infiltration in a rat model of SCI [88]. In addition to their production of NE [6, 48], infiltrating neutrophils contribute to inflammation via their production of IL-6 and TNF-α [42]. Systemic inflammation promotes vascular endothelial injury and results in organ dysfunction [60, 91]. Inflammatory cells and ECs express ANGPTs [4, 25, 53], suggesting that ANGPT signaling plays a central role in commencing and continuing the inflammatory response. Of note, ANGPT-1 and ANGPT-2 show dichotomous pro- and anti-inflammatory properties, with ANGPT-1 primarily considered anti-inflammatory and ANGPT-2 proinflammatory, which are influenced by other inflammatory mediators and proteolytic enzymes, such as NE [2, 9, 33, 44]. NE can regulate acute as well as chronic inflammation [21]. The data presented here demonstrate that SCI induces the expression of cytokines (TNF-α, IL-6, iNOS, and IL-1β) and chemokines (CCL-2 and CCL-3); the expression of the anti-inflammatory cytokine IL-10 decreased. The inhibition of NE with sivelestat treatment decreased these proinflammatory factors, including the expression of TNF-α, suggesting the beneficial role of NE inhibition on the ANGPT pathway after SCI. A previous report suggested that ANGPT-1 inhibits TNF-α-stimulated leukocyte transmigration [28], and ANGPT-2 sensitizes ECs to TNF-α and plays a crucial role in the induction of inflammation [26]. IL-6 is a proinflammatory cytokine that sharply increases in the acute phase after SCI and not only downregulates ANGPT-1 signaling [45] but also stimulates defective angiogenesis [29]. In the present study, treatment with sivelestat attenuated the SCI-induced IL-6 expression as well as that of iNOS, which is implicated in immune responses, inflammation, and apoptosis following SCI [79]. ANGPTs regulate vascular reactivity after hemorrhagic shock in rats through the Tie-2-nitric oxide pathway [95]. ANGPT-1 neutralizes the activity of proinflammatory factors on ECs, suppressing EC permeability induced by vascular endothelial growth factor, thrombin, bradykinin, and histamine [70, 72]. In addition, we observed that sivelestat augmented anti-inflammatory IL-10 while attenuating SCI-induced proinflammatory IL-1β, CCL-2, and CCL-3. Altogether, the evidence from this study supports the notion that acute sivelestat inhibition prevents the decrease in ANGPT-1 and acts as an anti-inflammatory in an experimental model of SCI.

NE is downstream in the humoral mediator network and is essential in vascular endothelial injury and increased permeability [78]. By contrast, ANGPT-1 suppresses vascular leakage/inflammation and expedites angiogenesis [51]. Previous studies showed that ANGPT-1 lowers vascular leakage by strengthening related endothelial molecules and regulating interendothelial adhesion. However, NE can protect the blood-brain barrier by increasing ANGPT-1 expression and EC survival in an animal model of focal ischemia [37]. BSCB disruption following SCI enables leukocytes, including neutrophils, to infiltrate the injured parenchyma, contributing to secondary injury [1, 32, 99]. As TJs provide a “barrier” or “fence” to regulate permeability and endothelial dysfunction [12], we speculated that NE is involved in the degradation of TJ proteins after SCI. We previously showed that the expression of the TJ proteins occludin and ZO-1 is decreased after moderate compression injury [48], suggesting a disruption of the BSCB. The decline in ANGPT-1 and increase in ANGPT-2 correspond with marked blood-brain barrier breakdown after brain injury [68]. Similarly, ANGPT-1 treatment was shown to attenuate BSCB permeability in an animal model of SCI [30, 35]. In the present study, the expression of the TJ proteins occludin and ZO-1 was reduced after SCI, suggesting BSCB disruption, which was prevented by treatment with sivelestat. Thus, specific inhibition of NE effectively prevented ANGPT-1 disruption and increased TJ protein expression after SCI.

After SCI, fibrotic scar tissue at the lesion site becomes rich in microglia, astroglia, and laminin and fibronectin forms in rodents and humans [77], which impedes axonal regeneration [50, 82]. NE damages fibronectins, laminins, and other matrix proteins, resulting in increased vascular permeability and haemorrhaging in tissues [36, 40]. Damaged ECs and the basal lamina deposited at the epicenter of the lesion diminish angiogenesis and are concurrent with cystic cavity formation. Sivelestat treatment reduced glial scar formation and secondary damage, facilitating neuronal regeneration at DPI-28. This was associated with a strong reduction in the amounts of microglia and astroglia at the injury site, as observed by IHC. However, SCI also induces inflammation, which contributes to fibrosis scarring in part via TGF-β signaling. Indeed, we observed a significant increase in TGF-β expression after SCI, which was attenuated in animals treated with sivelestat in accordance with the reduction in scar tissue formation.

Blood vessel density correlates with improved functional outcomes; hence sparing or regenerating the vasculature postinjury is desirable [43]. Although blood vessels tend to grow rapidly into the lesion site after SCI, there is substantial regression around 14 days postinjury [18, 56]. After nerve injury, PDGF-β expression increases [71], which leads to increased EC proliferation [11]. In the present study, sivelestat attenuated the increase in PDGF-β expression at the transcriptional level, as well as that for neuropilin1, whose expression in the spinal cord is normally low but is also upregulated after hemisection and dorsal column crush where it acts as an inhibitory molecule in regulating the organization of the sensory network [3]. Similarly, sivelestat attenuated the SCI-induced increase in PECAM-1, which acts as a mechanosensor in ECs [65] and whose localization to cell junctions is regulated by ANGPT-1 to maintain cellular integrity [28]. Another study found a significant loss of PECAM-1-positive cells at early time points (until day 3) after SCI, with a significant increase from 7 days onwards [94]. SCI also causes a robust decrease in vWF [59], which was observed in the present study. However, RECA-1 stained vessels could be readily identified within the injured spinal cord, suggesting that, although ECs are present, there is altered angiogenesis, which is prevented with NE inhibition.

As SCI can cause paralysis, altered motor coordination, and even neuropathic pain, we assessed these via behavioral tests in the animal model. A footprint analysis revealed reduced motor coordination in forepaw-hindpaw stepping after SCI. The functional impairment is likely influenced by the decrease in ANGPT-1 and related vascular dysfunction [15, 49, 73, 93], as exogenous administration of ANGPT-1 has shown favorable effects on both functional and vascular recovery [30, 35]. Accordingly, animals treated with sivelestat showed an increased ANGPT-1, marked recovery of gait and improved motor coordination compared with that of untreated injured animals. This functional recovery was also reflected by the increase in the BBB score in sivelestat-treated animals. The functional improvements were also accompanied by a reduction in SCI-induced hypersensitivity, an indicator of neuropathy, as assessed by hindpaw responses to stimulation with von Frey filaments. Thus functional recovery is a reflection of the increase in the regenerated area of the lesion. Previous reports suggest that enhanced axon regeneration correlates with functional recovery after SCI [19, 24]. Interestingly, intravenous injection of ANGPT-1 and αvβ3 integrin peptide results in almost complete recovery after SCI [30]. In the current study, the regeneration may have been facilitated by the increase in ANGPT-1, which promotes neurite outgrowth [47] and supports the differentiation of neural progenitor cells via the AKT pathway [8], as evidenced in the present study by the increase in AKT phosphorylation in sivelestat-treated animals. Furthermore, sivelestat treatment also maintained the levels of several neurotrophins (BDNF, NT-3, and NT-4) that are linked with EC survival [20] and are dramatically reduced in the adult spinal cord [16, 57]. In the current study, we treated sivelestat 1 h after injury. However additional studies are required to see whether sivelestat also work if treatment is delayed till 3~ 4 h after injury simulating the clinical settings. Secondly, owing to the short half-life of the sivelestat we treated it twice a day; it would be interesting to observe the effect of sivelestat as continuous infusion with a lower dose or increase the half-life or directly delivering the sivelestat into the spinal cord by several available approaches.

## Conclusions

In conclusion, our results indicate that NE expression is increased after SCI, resulting in a dissociation of ECs from microvessels, reduced ANGPT-1 expression, decreased angiogenesis, tissue damage, vascular destabilization, BSCB breakdown, and cell injury. The inhibition of NE via treatment with sivelestat significantly attenuated SCI-induced inflammation, prevented the decrease in ANGPT-1 expression, and attenuated the increase in ANGPT-2, BSCB breakdown, and cell injury. As a result, secondary damage, functional impairment, and neuropathic pain were reduced while vascular stabilization was promoted. Thus, NE inhibition could serve as a promising therapeutic strategy after SCI.

## Additional files

Additional file 1:  Method for pharmacokinetic study. (DOCX 14 kb)

Additional file 2: Primer sequences for the genes of interest and the reference genes. (DOCX 16 kb)

## References

[1] Abbott NJ, Ronnback L, Hansson E (2006). Astrocyte-endothelial interactions at the blood-brain barrier. Nat Rev Neurosci.

[2] Acarin L, Gonzalez B, Castellano B (2000). Neuronal, astroglial and microglial cytokine expression after an excitotoxic lesion in the immature rat brain. Eur J Neurosci.

[3] Agudo M, Robinson M, Cafferty W, Bradbury EJ, Kilkenny C, Hunt SP, McMahon SB (2005). Regulation of neuropilin 1 by spinal cord injury in adult rats. Mol Cell Neurosci.

[4] Ahmad S, Cudmore MJ, Wang K, Hewett P, Potluri R, Fujisawa T, Ahmed A (2010). Angiopoietin-1 induces migration of monocytes in a tie-2 and integrin-independent manner. Hypertension.

[5] Aikawa N, Kawasaki Y (2014). Clinical utility of the neutrophil elastase inhibitor sivelestat for the treatment of acute respiratory distress syndrome. Ther Clin Risk Manag.

[6] Ankeny DP, Popovich PG (2009). Mechanisms and implications of adaptive immune responses after traumatic spinal cord injury. Neuroscience.

[7] Aube B, Levesque SA, Pare A, Chamma E, Kebir H, Gorina R, Lecuyer MA, Alvarez JI, De Koninck Y, Engelhardt B, Prat A, Cote D, Lacroix S (2014). Neutrophils mediate blood-spinal cord barrier disruption in demyelinating neuroinflammatory diseases. J Immunol.

[8] Bai Y, Cui M, Meng Z, Shen L, He Q, Zhang X, Chen F, Xiao J (2009). Ectopic expression of angiopoietin-1 promotes neuronal differentiation in neural progenitor cells through the Akt pathway. Biochem Biophys Res Commun.

[9] Bartholdi D, Schwab ME (1997). Expression of pro-inflammatory cytokine and chemokine mRNA upon experimental spinal cord injury in mouse: an in situ hybridization study. Eur J Neurosci.

[10] Basso DM, Beattie MS, Bresnahan JC (1995). A sensitive and reliable locomotor rating scale for open field testing in rats. J Neurotrauma.

[11] Battegay EJ, Rupp J, Iruela-Arispe L, Sage EH, Pech M (1994). PDGF-BB modulates endothelial proliferation and angiogenesis in vitro via PDGF beta-receptors. J Cell Biol.

[12] Bazzoni G, Dejana E (2004). Endothelial cell-to-cell junctions: molecular organization and role in vascular homeostasis. Physiol Rev.

[13] Beck H, Acker T, Wiessner C, Allegrini PR, Plate KH (2000). Expression of angiopoietin-1, angiopoietin-2, and tie receptors after middle cerebral artery occlusion in the rat. Am J Pathol.

[14] Belaaouaj A, McCarthy R, Baumann M, Gao Z, Ley TJ, Abraham SN, Shapiro SD (1998). Mice lacking neutrophil elastase reveal impaired host defense against gram negative bacterial sepsis. Nat Med.

[15] Benton RL, Maddie MA, Worth CA, Mahoney ET, Hagg T, Whittemore SR (2008). Transcriptomic screening of microvascular endothelial cells implicates novel molecular regulators of vascular dysfunction after spinal cord injury. J Cereb Blood Flow Metab.

[16] Blesch A, Yang H, Weidner N, Hoang A, Otero D (2004). Axonal responses to cellularly delivered NT-4/5 after spinal cord injury. Mol Cell Neurosci.

[17] Bonin RP, Bories C, De Koninck Y (2014). A simplified up-down method (SUDO) for measuring mechanical nociception in rodents using von Frey filaments. Mol Pain.

[18] Casella GT, Marcillo A, Bunge MB, Wood PM (2002). New vascular tissue rapidly replaces neural parenchyma and vessels destroyed by a contusion injury to the rat spinal cord. Exp Neurol.

[19] Coumans JV, Lin TT-S, Dai HN, MacArthur L, McAtee M, Nash C, Bregman BS (2001). Axonal regeneration and functional recovery after complete spinal cord transection in rats by delayed treatment with transplants and neurotrophins. J Neurosci.

[20] Donovan MJ, Lin MI, Wiegn P, Ringstedt T, Kraemer R, Hahn R, Wang S, Ibañez CF, Rafii S, Hempstead BL (2000). Brain derived neurotrophic factor is an endothelial cell survival factor required for intramyocardial vessel stabilization. Development.

[21] Doring G (1994). The role of neutrophil elastase in chronic inflammation. Am J Respir Crit Care Med.

[22] Durham-Lee JC, Wu Y, Mokkapati VU, Paulucci-Holthauzen AA, Nesic O (2012). Induction of angiopoietin-2 after spinal cord injury. Neuroscience.

[23] Engelhardt B, Coisne C (2011). Fluids and barriers of the CNS establish immune privilege by confining immune surveillance to a two-walled castle moat surrounding the CNS castle. Fluids Barriers CNS.

[24] Fawcett JW (2009). Recovery from spinal cord injury: regeneration, plasticity and rehabilitation. Brain.

[25] Feistritzer C, Mosheimer BA, Sturn DH, Bijuklic K, Patsch JR, Wiedermann CJ (2004). Expression and function of the angiopoietin receptor Tie-2 in human eosinophils. J Allergy Clin Immunol.

[26] Fiedler U, Reiss Y, Scharpfenecker M, Grunow V, Koidl S, Thurston G, Gale NW, Witzenrath M, Rosseau S, Suttorp N (2006). Angiopoietin-2 sensitizes endothelial cells to TNF-α and has a crucial role in the induction of inflammation. Nat Med.

[27] Figley SA, Khosravi R, Legasto JM, Tseng YF, Fehlings MG (2014). Characterization of vascular disruption and blood-spinal cord barrier permeability following traumatic spinal cord injury. J Neurotrauma.

[28] Gamble JR, Drew J, Trezise L, Underwood A, Parsons M, Kasminkas L, Rudge J, Yancopoulos G, Vadas MA (2000). Angiopoietin-1 is an antipermeability and anti-inflammatory agent in vitro and targets cell junctions. Circ Res.

[29] Gopinathan G, Milagre C, Pearce OM, Reynolds LE, Hodivala-Dilke K, Leinster DA, Zhong H, Hollingsworth RE, Thompson R, Whiteford JR (2015). Interleukin-6 stimulates defective angiogenesis. Cancer Res.

[30] Han S, Arnold SA, Sithu SD, Mahoney ET, Geralds JT, Tran P, Benton RL, Maddie MA, D'Souza SE, Whittemore SR, Hagg T (2010). Rescuing vasculature with intravenous angiopoietin-1 and alpha v beta 3 integrin peptide is protective after spinal cord injury. Brain.

[31] Hansen TM, Moss AJ, Brindle NP (2008). Vascular endothelial growth factor and angiopoietins in neurovascular regeneration and protection following stroke. Curr Neurovasc Res.

[32] Hawkins BT, Davis TP (2005). The blood-brain barrier/neurovascular unit in health and disease. Pharmacol Rev.

[33] Hayashi M, Ueyama T, Nemoto K, Tamaki T, Senba E (2000). Sequential mRNA expression for immediate early genes, cytokines, and neurotrophins in spinal cord injury. J Neurotrauma.

[34] Hermant B, Bibert S, Concord E, Dublet B, Weidenhaupt M, Vernet T, Gulino-Debrac D (2003). Identification of proteases involved in the proteolysis of vascular endothelium cadherin during neutrophil transmigration. J Biol Chem.

[35] Herrera JJ, Sundberg LM, Zentilin L, Giacca M, Narayana PA (2010). Sustained expression of vascular endothelial growth factor and angiopoietin-1 improves blood-spinal cord barrier integrity and functional recovery after spinal cord injury. J Neurotrauma.

[36] Houtz PK, Jones PD, Aronson NE, Richardson LM, Lai-Fook SJ (2004). Effect of pancreatic and leukocyte elastase on hydraulic conductivity in lung interstitial segments. J Appl Physiol (1985).

[37] Ikegame Y, Yamashita K, Hayashi S, Yoshimura S, Nakashima S, Iwama T (2010). Neutrophil elastase inhibitor prevents ischemic brain damage via reduction of vasogenic edema. Hypertens Res.

[38] Inoue N, Oka N, Kitamura T, Shibata K, Itatani K, Tomoyasu T, Miyaji K (2013). Neutrophil elastase inhibitor sivelestat attenuates perioperative inflammatory response in pediatric heart surgery with cardiopulmonary bypass. Int Heart J.

[39] Ionescu CV, Cepinskas G, Savickiene J, Sandig M, Kvietys PR (2003). Neutrophils induce sequential focal changes in endothelial adherens junction components: role of elastase. Microcirculation.

[40] Ishikawa N, Oda M, Kawaguchi M, Tsunezuka Y, Watanabe G (2003). The effects of a specific neutrophil elastase inhibitor (ONO-5046) in pulmonary ischemia-reperfusion injury. Transpl Int.

[41] Iwamoto S, Higashi A, Ueno T, Goto M, Iguro Y, Sakata R (2009). Protective effect of sivelestat sodium hydrate (ONO-5046) on ischemic spinal cord injury. Interact Cardiovasc Thorac Surg.

[42] Jablonska E, Kiluk M, Markiewicz W, Piotrowski L, Grabowska Z, Jablonski J (2001). TNF-alpha, IL-6 and their soluble receptor serum levels and secretion by neutrophils in cancer patients. Arch Immunol Ther Exp (Warsz).

[43] Kaneko S, Iwanami A, Nakamura M, Kishino A, Kikuchi K, Shibata S, Okano HJ, Ikegami T, Moriya A, Konishi O (2006). A selective Sema3A inhibitor enhances regenerative responses and functional recovery of the injured spinal cord. Nat Med.

[44] Kawabata K, Hagio T, Matsuoka S (2002). The role of neutrophil elastase in acute lung injury. Eur J Pharmacol.

[45] Kayakabe K, Kuroiwa T, Sakurai N, Ikeuchi H, Kadiombo AT, Sakairi T, Matsumoto T, Maeshima A, Hiromura K, Nojima Y (2012) Interleukin-6 promotes destabilized angiogenesis by modulating angiopoietin expression in rheumatoid arthritis. Rheumatology. 10.1093/rheumatology/kes093. 10.1093/rheumatology/kes093 22596210

[46] Kim H, Lee JM, Park JS, Jo SA, Kim YO, Kim CW, Jo I (2008). Dexamethasone coordinately regulates angiopoietin-1 and VEGF: a mechanism of glucocorticoid-induced stabilization of blood-brain barrier. Biochem Biophys Res Commun.

[47] Kosacka J, Figiel M, Engele J, Hilbig H, Majewski M, Spanel-Borowski K (2005). Angiopoietin-1 promotes neurite outgrowth from dorsal root ganglion cells positive for Tie-2 receptor. Cell Tissue Res.

[48] Kumar H, Jo MJ, Choi H, Muttigi MS, Shon S, Kim BJ, Lee SH, Han IB (2017) Matrix Metalloproteinase-8 Inhibition Prevents Disruption of Blood-Spinal Cord Barrier and Attenuates Inflammation in Rat Model of Spinal Cord Injury. Mol Neurobiol. 10.1007/s12035-017-0509-3 10.1007/s12035-017-0509-3 28421532

[49] Kumar H, Ropper AE, Lee S-H, Han I (2017) Propitious Therapeutic Modulators to Prevent Blood-Spinal Cord Barrier Disruption in Spinal Cord Injury. Mol Neurobiol 54(5):3578–3590. 10.1007/s12035-016-9910-6 10.1007/s12035-016-9910-6 27194298

[50] Leal-Filho MB (2011). Spinal cord injury: From inflammation to glial scar. Surg Neurol Int.

[51] Lee HS, Han J, Bai HJ, Kim KW (2009). Brain angiogenesis in developmental and pathological processes: regulation, molecular and cellular communication at the neurovascular interface. FEBS J.

[52] Lee SM, Rosen S, Weinstein P, van Rooijen N, Noble-Haeusslein LJ (2011). Prevention of both neutrophil and monocyte recruitment promotes recovery after spinal cord injury. J Neurotrauma.

[53] Lemieux C, Maliba R, Favier J, Theoret JF, Merhi Y, Sirois MG (2005). Angiopoietins can directly activate endothelial cells and neutrophils to promote proinflammatory responses. Blood.

[54] Ling X, Liu D (2007). Temporal and spatial profiles of cell loss after spinal cord injury: Reduction by a metalloporphyrin. J Neurosci Res.

[55] Liu XZ, Xu XM, Hu R, Du C, Zhang SX, McDonald JW, Dong HX, Wu YJ, Fan GS, Jacquin MF, Hsu CY, Choi DW (1997). Neuronal and glial apoptosis after traumatic spinal cord injury. J Neurosci.

[56] Loy DN, Crawford CH, Darnall JB, Burke DA, Onifer SM, Whittemore SR (2002). Temporal progression of angiogenesis and basal lamina deposition after contusive spinal cord injury in the adult rat. J Comp Neurol.

[57] Maisonpierre PC, Belluscio L, Friedman B, Alderson RF, Wiegand SJ, Furth ME, Lindsay RM, Yancopoulos GD (1990). NT-3, BDNF, and NGF in the developing rat nervous system: parallel as well as reciprocal patterns of expression. Neuron.

[58] Man S, Ubogu EE, Ransohoff RM (2007). Inflammatory cell migration into the central nervous system: a few new twists on an old tale. Brain Pathol.

[59] Matsushita T, Lankford KL, Arroyo EJ, Sasaki M, Neyazi M, Radtke C, Kocsis JD (2015). Diffuse and persistent blood–spinal cord barrier disruption after contusive spinal cord injury rapidly recovers following intravenous infusion of bone marrow mesenchymal stem cells. Exp Neurol.

[60] McGill SN, Ahmed NA, Christou NV (1998). Endothelial cells: role in infection and inflammation. World J Surg.

[61] Nag S, Papneja T, Venugopalan R, Stewart DJ (2005). Increased angiopoietin2 expression is associated with endothelial apoptosis and blood-brain barrier breakdown. Lab Invest.

[62] Nakatani K, Takeshita S, Tsujimoto H, Kawamura Y, Sekine I (2001). Inhibitory effect of serine protease inhibitors on neutrophil-mediated endothelial cell injury. J Leukoc Biol.

[63] National Research Council (2011) Guide for the care and use of laboratory animals. Eighth Edition. Washington, DC: National Academies Press.

[64] Neirinckx V, Coste C, Franzen R, Gothot A, Rogister B, Wislet S (2014). Neutrophil contribution to spinal cord injury and repair. J Neuroinflammation.

[65] Newman PJ (1999). Switched at birth: a new family for PECAM-1. J Clin Invest.

[66] Noble LJ, Mautes AE, Hall JJ (1996) Characterization of the microvascular glycocalyx in normal and injured spinal cord in the rat. J Comp Neurol 376:542–556. 10.1002/(SICI)1096-9861(19961223)376:4<542::AID-CNE4>3.0.CO;2-1 10.1002/(SICI)1096-9861(19961223)376:4<542::AID-CNE4>3.0.CO;2-1 8978469

[67] Noble LJ, Wrathall JR (1989). Distribution and time course of protein extravasation in the rat spinal cord after contusive injury. Brain Res.

[68] Nourhaghighi N, Teichert-Kuliszewska K, Davis J, Stewart DJ, Nag S (2003). Altered expression of angiopoietins during blood-brain barrier breakdown and angiogenesis. Lab Invest.

[69] Okajima K, Harada N, Uchiba M, Mori M (2004). Neutrophil elastase contributes to the development of ischemia-reperfusion-induced liver injury by decreasing endothelial production of prostacyclin in rats. Am J Physiol Gastrointest Liver Physiol.

[70] Oubaha M, Gratton JP (2009). Phosphorylation of endothelial nitric oxide synthase by atypical PKC zeta contributes to angiopoietin-1-dependent inhibition of VEGF-induced endothelial permeability in vitro. Blood.

[71] Oya T, Zhao YL, Takagawa K, Kawaguchi M, Shirakawa K, Yamauchi T, Sasahara M (2002). Platelet-derived growth factor-b expression induced after rat peripheral nerve injuries. Glia.

[72] Pizurki L, Zhou Z, Glynos K, Roussos C, Papapetropoulos A (2003). Angiopoietin-1 inhibits endothelial permeability, neutrophil adherence and IL-8 production. Br J Pharmacol.

[73] Popovich PG, Horner PJ, Mullin BB, Stokes BT (1996). A quantitative spatial analysis of the blood-spinal cord barrier. I. Permeability changes after experimental spinal contusion injury. Exp Neurol.

[74] Ritz MF, Graumann U, Gutierrez B, Hausmann O (2010). Traumatic spinal cord injury alters angiogenic factors and TGF-beta1 that may affect vascular recovery. Curr Neurovasc Res.

[75] Ropper AE, Zeng X, Anderson JE, Yu D, Han I, Haragopal H, Teng YD (2015). An efficient device to experimentally model compression injury of mammalian spinal cord. Exp Neurol.

[76] Rossignol S, Schwab M, Schwartz M, Fehlings MG (2007). Spinal cord injury: time to move?. J Neurosci.

[77] Ruschel J, Hellal F, Flynn KC, Dupraz S, Elliott DA, Tedeschi A, Bates M, Sliwinski C, Brook G, Dobrindt K, Peitz M, Brustle O, Norenberg MD, Blesch A, Weidner N, Bunge MB, Bixby JL, Bradke F (2015). Axonal regeneration. Systemic administration of epothilone B promotes axon regeneration after spinal cord injury. Science.

[78] Russell JA (2006). Management of sepsis. N Engl J Med.

[79] Satake K, Matsuyama Y, Kamiya M, Kawakami H, Iwata H, Adachi K, Kiuchi K (2000). Nitric oxide via macrophage iNOS induces apoptosis following traumatic spinal cord injury. Brain Res Mol Brain Res.

[80] Scali M, Begenisic T, Mainardi M, Milanese M, Bonifacino T, Bonanno G, Sale A, Maffei L (2013). Fluoxetine treatment promotes functional recovery in a rat model of cervical spinal cord injury. Sci Rep.

[81] Semple BD, Trivedi A, Gimlin K, Noble-Haeusslein LJ (2015). Neutrophil elastase mediates acute pathogenesis and is a determinant of long-term behavioral recovery after traumatic injury to the immature brain. Neurobiol Dis.

[82] Silver J, Miller JH (2004). Regeneration beyond the glial scar. Nat Rev Neurosci.

[83] Smedly LA, Tonnesen MG, Sandhaus RA, Haslett C, Guthrie LA, Johnston RB, Henson PM, Worthen GS (1986). Neutrophil-mediated injury to endothelial cells. Enhancement by endotoxin and essential role of neutrophil elastase. J Clin Invest.

[84] Taoka Y, Okajima K, Murakami K, Johno M, Naruo M (1998). Role of neutrophil elastase in compression-induced spinal cord injury in rats. Brain Res.

[85] Tator CH, Koyanagi I (1997). Vascular mechanisms in the pathophysiology of human spinal cord injury. J Neurosurg.

[86] Thomas M, Augustin HG (2009). The role of the Angiopoietins in vascular morphogenesis. Angiogenesis.

[87] Thurston G, Rudge JS, Ioffe E, Zhou H, Ross L, Croll SD, Glazer N, Holash J, McDonald DM, Yancopoulos GD (2000). Angiopoietin-1 protects the adult vasculature against plasma leakage. Nat Med.

[88] Tonai T, Shiba K, Taketani Y, Ohmoto Y, Murata K, Muraguchi M, Ohsaki H, Takeda E, Nishisho T (2001). A neutrophil elastase inhibitor (ONO-5046) reduces neurologic damage after spinal cord injury in rats. J Neurochem.

[89] Travis J (1988). Structure, function, and control of neutrophil proteinases. Am J Med.

[90] Tsuboko Y, Takeda S, Mii S, Nakazato K, Tanaka K, Uchida E, Sakamoto A (2012). Clinical evaluation of sivelestat for acute lung injury/acute respiratory distress syndrome following surgery for abdominal sepsis. Drug Des Devel Ther.

[91] Ueno H, Hirasawa H, Oda S, Shiga H, Nakanishi K, Matsuda K (2002). Coagulation/fibrinolysis abnormality and vascular endothelial damage in the pathogenesis of thrombocytopenic multiple organ failure. Crit Care Med.

[92] Valenzuela DM, Griffiths JA, Rojas J, Aldrich TH, Jones PF, Zhou H, McClain J, Copeland NG, Gilbert DJ, Jenkins NA, Huang T, Papadopoulos N, Maisonpierre PC, Davis S, Yancopoulos GD (1999). Angiopoietins 3 and 4: diverging gene counterparts in mice and humans. Proc Natl Acad Sci U S A.

[93] Whetstone WD, Hsu JY, Eisenberg M, Werb Z, Noble-Haeusslein LJ (2003). Blood-spinal cord barrier after spinal cord injury: relation to revascularization and wound healing. J Neurosci Res.

[94] Whetstone WD, Hsu JYC, Eisenberg M, Werb Z, Noble-Haeusslein LJ (2003). Blood-spinal cord barrier after spinal cord injury: Relation to revascularization and wound healing. J Neurosci Res.

[95] Xu J, Lan D, Li T, Yang G, Liu L (2012). Angiopoietins regulate vascular reactivity after haemorrhagic shock in rats through the Tie2-nitric oxide pathway. Cardiovasc Res.

[96] Yang JJ, Kettritz R, Falk RJ, Jennette JC, Gaido ML (1996). Apoptosis of endothelial cells induced by the neutrophil serine proteases proteinase 3 and elastase. Am J Pathol.

[97] Young RE, Thompson RD, Larbi KY, La M, Roberts CE, Shapiro SD, Perretti M, Nourshargh S (2004). Neutrophil elastase (NE)-deficient mice demonstrate a nonredundant role for NE in neutrophil migration, generation of proinflammatory mediators, and phagocytosis in response to zymosan particles in vivo. J Immunol.

[98] Zacharek A, Chen J, Cui X, Li A, Li Y, Roberts C, Feng Y, Gao Q, Chopp M (2007). Angiopoietin1/Tie2 and VEGF/Flk1 induced by MSC treatment amplifies angiogenesis and vascular stabilization after stroke. J Cereb Blood Flow Metab.

[99] Zlokovic BV (2008). The blood-brain barrier in health and chronic neurodegenerative disorders. Neuron.

