# Supplementary material for: Neutrophil elastase inhibition effectively rescued angiopoietin-1 decrease and inhibits glial scar after spinal cord injury
Source: Acta Neuropathol Commun. 2018 Aug 7;6:73. doi: 10.1186/s40478-018-0576-3 (PMC6080383; doi:10.1186/s40478-018-0576-3)
Supplement: Supplementary file 1 — Method for pharmacokinetic study. (DOCX 14 kb) [file 40478_2018_576_MOESM1_ESM.docx]

Additional file 1

**Pharmacokinetic Study**

*Reagents and chemicals*

Trifluoroacetic acid (TFA) and formic acid were purchased from Sigma-Aldrich (St, Louis, MO, USA). HPLC grade acetonitrile and methanol were purchased from Burdick & Jackson (Muskegon, MI, USA). Water filtered on-site with a Millipore Milli-Q system (Millipore, Billerica, MA) was used for the study.

*Stock and Sample Preparation*

A stock solution of sivelestat and propyl parahydroxybenzoate as an internal standard was prepared at 1mg/mL in water and methanol. They were stored in -20°C before use. A simple liquid-liquid extraction process was followed for extraction of sivelestat from rat plasma, brain and spinal cord. To an aliquot of 100 μL plasma, 100 μL IS solution and 1 mL 2N HCl solution was added and mixed. After the addition of 2 mL of ethyl acetate, the mixture was vortexed, followed by centrifugation for 10 min at 3000 rpm. The organic layer was evaporated to dryness at 40 °C using a gentle stream of nitrogen. The residue was reconstituted in 100 μL the mobile phase and was injected onto HPLC and LC-MS/MS system.

*Chromatographic conditions*

Separation and detection were carried out on a Shimazu high-performance liquid chromatography (HPLC) system (Shimazu, Japan) equipped with an autosampler and PDA detector for separation of sivelestat in plasma. The separation was performed on a Luna C18 reverse-phase HPLC column (150 x 4.6mm, Phenomenex, USA) by isocratic elution with a mixture 0.75% TFA in water and 0.75% TFA in acetonitrile (6:4, v/v). The UV wavelength, flow rate, and injection volume were 254 nm, 1.0 mL/min and 20 μl respectively.

Separation of sivelestat in the brain and spinal cord was carried out on a Shimazu high-performance liquid chromatography (HPLC) system (Shimazu, Japan). The separation was performed on a Waters C18 reverse-phase HPLC column (10 x 2.0 mm, Waters, USA) by gradient elution with a mixture 0.1% formic acid in water and 0.1% formic acid in acetonitrile (55:45, v/v). The flow rate and injection volumes were 0.5 mL/min and 2 μl respectively. Quantitation was achieved by MS/MS detection in negative ion mode for sivelestat and IS using an API-4000 mass spectrometer (AB Sciex, USA). Detection of the ions was performed in the multiple reaction monitoring mode, monitoring the transition of the m/z 443 precursor ion to the m/z 397 product ion for sivelestat and m/z 179 precursor ion to the m/z 93 product ion for IS.
