# Supplementary material for: Neutrophil elastase inhibition effectively rescued angiopoietin-1 decrease and inhibits glial scar after spinal cord injury
Source: Acta Neuropathol Commun. 2018 Aug 7;6:73. doi: 10.1186/s40478-018-0576-3 (PMC6080383; doi:10.1186/s40478-018-0576-3)
Supplement: Supplementary file 2 — Primer sequences for the genes of interest and the reference genes. (DOCX 16 kb) [file 40478_2018_576_MOESM2_ESM.docx]

Additional file 2: Table S1

| **Oligo Name** | **Sequence (5’ --------> 3’)** | |  |
| --- | --- | --- | --- |
| **Occludin (Rat)** | | Forward | ATCTAGAGCCTGGAGCAACG |
|  |  | Reverse | GTCAAGGCTCCCAAGACAAG |
| **ZO-1 (Rat)** | | Forward | AGTTCTGCCCTCAGCTACCA |
|  |  | Reverse | GCTTAAAGCTGGCAGTGTC |
| **iNOS (Rat)** | | Forward | ACCATGGAGCATCCCAAGT |
|  |  | Reverse | CAGCGCATACCACTTCAGC |
| **TNF-α (Rat)** | | Forward | ACTGAACTTCGGGGTGATTG |
|  |  | Reverse | GCTTGGTGGTTTGCTACGAC |
| **IL-6 (Rat)** | | Forward | TGATGGATGCTTCCAAACTG |
|  |  | Reverse | GAGCATTGGAAGTTGGGGTA |
| **IL-1β (Rat)** | | Forward | TGCTGATGTACCAGTTGGGG |
|  |  | Reverse | CTCCATGAGCTTTGTACAAG |
| **IL-10 (Rat)** | | Forward | TGGACAACATACTGCTGACAG |
|  |  | Reverse | GGTAAAACTTGATCATTTCTGACAAG |
| **CCL-2 (Rat)** | | Forward | GTGCTGACCCCAATAAGGAA |
|  |  | Reverse | TGAGGTGGTTGTGGAAAAGA |
| **CCL-3 (Rat)** | | Forward | GCGCTCTGGAACGAAGTCT |
|  |  | Reverse | GAATTTGCCGTCCATAGGAG |
| **Mac-1 (Rat)** | | Forward | CTGCCTCAGGGATCCGTAAAG |
|  |  | Reverse | CCTCTGCCTCAGGAATGACATC |
| **ANGPT-1 (Rat)** | | Forward | TTTAGATTGGAAGGGCCACA |
|  |  | Reverse | ATGCGCCCTTATGCTAACAG |
| **ANGPT-2 (Rat)** | | Forward | CCCACTTCTGAGCTTCACATC |
|  |  | Reverse | CATAGGAGGAAACCTGTTCACC |
| **Neutrophil elastase (Rat)** | | Forward | GCCTTGCTCCTGGTCTGTC |
|  |  | Reverse | ACACCATGAAGGGCCAAG |
| **Neuropilin-1 (RAT)** | | Forward | CATAGTGGGCTCGGACTGA |
|  |  | Reverse | GGGTCCAGCTGTAGGCACT |
| **TGF-β (Rat)** | | Forward | GCAACAACGCAATCTATGAC |
|  |  | Reverse | CCTGTATTCCGTCTCCTT |
| **PECAM (Rat)** | | Forward | TCCCCACCCAAAGTAGCA |
|  |  | Reverse | CAAGGCAGGAGGGATTTACA |
| **PDGF- β (Rat)** | | Forward | GCCAACTTCCTGGTGTGG |
|  |  | Reverse | CACAATTTCGATCTTTCTCACCT |
| **GFAP (Rat)** | | Forward | GGTGGAGAGGGACAATCTCA |
|  |  | Reverse | CCAGCTGCTCCTGGAGTTCT |
| **Iba-1 (Rat)** | | Forward | CAGACGCACCCTCTGATGT |
|  |  | Reverse | CTCCAAGAATGGGGTGAGC |
| **GAPDH (Rat)** | | Forward | CAACTCCCTCAAGATTGTCAGCAA |
|  |  | Reverse | GGCATGGACTGTGGTCATGA |
| **ANGPT-1 (Human)** | | Forward | GAC AGA TGT TGA GAC CCA GGT A |
|  |  | Reverse | TCT CTA GCT TGT AGG TGG ATA ATG AA |
| **ANGPT-2 (Human)** | | Forward | TGC AAA TGT TCA CAA ATG CTA A |
|  |  | Reverse | AAG TTG GAA GGA CCA CAT GC |
| **18 S (Human)** | | Forward | GTA ACC CGT TGA ACC CCA TT |
|  |  | Reverse | CCA TCC AAT CGG TAG TAG CG |
| ZO-1- Zona occludens; iNOS- Inducible Nitric Oxide; TNF-α - Tumor necrosis factor-alpha; IL-6-Interleukin 6; IL-1β- Interleukin-1 beta; IL-10 - Interleukin-10; CCL-2 - C-C motif chemokine ligand-2; CCL-3 - C-C motif chemokine ligand-3; Mac-1 - Macrophage-1 antigen; ANGPT-1 - Angiopoietin-1; ANGPT-2 - Angiopoietin-2; TGF-β - Transforming growth factor beta 1; PECAM- Platelet endothelial cell adhesion molecule; PDGF- β- Platelet-derived growth factor subunit B; GFAP - Glial fibrillary acidic protein; Iba-1 - ionized calcium-binding adapter molecule 1; GAPDH - Glyceraldehyde 3-phosphate dehydrogenase. | | | |
